# A Forward-Reverse Brascamp-Lieb Inequality: Entropic Duality and Gaussian Optimality

**DOI:** 10.3390/e20060418

**Published:** 2018-05-30

**Authors:** Jingbo Liu, Thomas A. Courtade, Paul W. Cuff, Sergio Verdú

**Affiliations:** 1 Department of Electrical Engineering, Princeton University, Princeton, NJ 08544, USA; 2 Department of Electrical Engineering and Computer Sciences, University of California, Berkeley, CA 94720-1770, USA; 3 Renaissance Technologies, LLC 600 Route 25A East Setauket, New York, NY 11733, USA

**Keywords:** Brascamp-Lieb inequality, hypercontractivity, functional-entropic duality, Gaussian optimality, network information theory, image size characterization

## Abstract

Inspired by the forward and the reverse channels from the image-size characterization problem in network information theory, we introduce a functional inequality that unifies both the Brascamp-Lieb inequality and Barthe’s inequality, which is a reverse form of the Brascamp-Lieb inequality. For Polish spaces, we prove its equivalent entropic formulation using the Legendre-Fenchel duality theory. Capitalizing on the entropic formulation, we elaborate on a “doubling trick” used by Lieb and Geng-Nair to prove the Gaussian optimality in this inequality for the case of Gaussian reference measures.

## 1. Introduction

The Brascamp-Lieb inequality and its reverse [1] concern the optimality of Gaussian functions in a certain type of integral inequality. (Not to be confused with the “variance Brascamp-Lieb inequality” (cf. [2,3,4]), which generalizes the Poincaré inequality). These inequalities have been generalized in various ways since their discovery, nearly 40 years ago. A modern formulation due to Barthe [5] may be stated as follows:

**Brascamp-Lieb Inequality and Its Reverse**  ([5] Theorem 1). *Let E, $E_{1}$, …, $E_{m}$ be Euclidean spaces and $B_{i}:E\to E_{i}$ be linear maps. Let ${\left(c_{i}\right)}_{i=1}^{m}$ and D be positive real numbers. Then, the Brascamp-Lieb inequality:*

(1) $$\begin{array}{c} \int \prod_{i=1}^{m}f_{i}^{c_{i}}\left(B_{i}x\right)\;dx\leq D\prod_{i=1}^{m}{\left(\int f_{i}\left(x_{i}\right)dx_{i}\right)}^{c_{i}}, \end{array}$$

*for all nonnegative measurable functions $f_{i}$ on $E_{i}$, i=1,…,m, holds if and only if it holds whenever $f_{i}$, i=1,…,m are centered Gaussian functions (a centered Gaussian function is of the form $x\mapsto \exp(r-x^{⊤}Ax)$, where A is a positive semidefinite matrix and r∈R). Similarly, for F a positive real number, the reverse Brascamp-Lieb inequality, also known as Barthe’s inequality ($B_{i}^{*}$ denotes the adjoint of $B_{i}$),*

(2) $$\begin{array}{c} \int \underset{\left(y_{i}\right):\sum_{i=1}^{m}c_{i}B_{i}^{*}y_{i}=x}{\sup}\prod_{i=1}^{m}f_{i}^{c_{i}}\left(y_{i}\right)\;dx\geq F\prod_{i=1}^{m}{\left(\int f_{i}\left(y_{i}\right)dy_{i}\right)}^{c_{i}}, \end{array}$$

*for all nonnegative measurable functions $f_{i}$ on $E_{i}$, i=1,…,m, holds if and only if it holds for all centered Gaussian functions.*

For surveys on the history of both the Brascamp-Lieb inequality and Barthe’s inequality and their applications, see, e.g., [6,7]. The Brascamp-Lieb inequality can be seen as a generalization of several other inequalities, including Hölder’s inequality, the sharp Young inequality, the Loomis-Whitney inequality, the entropy power inequality (cf. [6] or the survey paper [8]), hypercontractivity and the logarithmic Sobolev inequality [9]. Furthermore, the Prékopa-Leindler inequality can be seen as a special case of Barthe’s inequality. Due in part to their utility in establishing impossibility bounds, these functional inequalities have attracted much attention in information theory [10,11,12,13,14,15,16,17], theoretical computer science [18,19,20,21,22] and statistics [23,24,25,26,27,28], to name only a small subset of the literature. Over the years, various proofs of these inequalities have been proposed [1,29,30,31,32,33,34]. Among these, Lieb’s elegant proof [29], which is very close to one of the techniques that will be used in this paper, employs a doubling trick that capitalizes on the rotational invariance property of the Gaussian function: if *f* is a one-dimensional Gaussian function, then:

(3) $$\begin{array}{c} f\left(x\right)f\left(y\right)=f\left(\frac{x-y}{\sqrt{2}}\right)f\left(\frac{x+y}{\sqrt{2}}\right). \end{array}$$

Since (1) and (2) have the same structure modulo the direction of the inequality, a common viewpoint is to consider (1) and (2) as dual inequalities. This viewpoint successfully captures the geometric aspects of (1) and (2). Indeed, it is known that:

(4) $$\begin{array}{c} D\cdot F=1 \end{array}$$

as long as D,F<∞ [5]. Moreover, both *D* and *F* are equal to one under Ball’s geometric condition [35]: $E_{1}$, …, $E_{m}$ are dimension one, and:

(5) $$\begin{array}{c} \sum_{i=1}^{m}c_{i}B_{i}B_{i}^{*}=I \end{array}$$

is the identity matrix. While fruitful, this “dual” viewpoint does not fully explain the asymmetry between the forward and the reverse inequalities: there is a sup in (2), but not in (1).

This paper explores a different viewpoint. In particular, we propose a single inequality that unifies (1) and (2). Accordingly, we should reverse both sides of (2) to make the inequality sign consistent with (1). To be concrete, let us first observe that (1) and (2) can be respectively restated in the following more symmetrical forms (with changes of certain symbols):

- For all nonnegative functions *g* and $f_{1},\ldots ,f_{m}$ such that:
  (6) $$\begin{array}{c} g\left(x\right)\leq \prod_{i=1}^{m}f_{j}^{c_{j}}\left(B_{j}x\right),\;\forall x, \end{array}$$
  we have:
  (7) $$\begin{array}{c} \int_{E}g\leq D\prod_{j=1}^{m}{\left(\int_{E_{j}}f_{j}\right)}^{c_{j}}. \end{array}$$
- For all nonnegative measurable functions $g_{1},\ldots g_{l}$ and *f* such that:
  (8) $$\begin{array}{c} \prod_{i=1}^{l}g_{i}^{b_{i}}\left(z_{i}\right)\leq f\left(\sum_{i=1}^{l}b_{i}B_{i}^{*}z_{i}\right),\;\forall z_{1},\ldots ,z_{l}, \end{array}$$
  we have:
  (9) $$\begin{array}{c} \prod_{i=1}^{l}{\left(\int_{E_{i}}g_{i}\right)}^{b_{i}}\leq D\int_{E}f. \end{array}$$

Note that in both cases, the optimal choice of one function (*f* or *g*) can be explicitly computed from the constraints, hence the conventional formulations in (1) and (2). Generalizing further, we can consider the following problem: Let X, $Y_{1},\ldots ,Y_{m}$, $Z_{1},\ldots ,Z_{l}$ be measurable spaces. Consider measurable maps $\phi_{j}:X\to Y_{j}$, j=1,…,m and $\psi :X\to Z_{i}$, i=1,…,l. Let $b_{1},\ldots ,b_{l}$ and $c_{1},\ldots ,c_{m}$ be nonnegative real numbers. Let $\nu_{1},\ldots ,\nu_{l}$ be measures on $Z_{1},\ldots ,Z_{l}$ and $\mu_{1},\ldots ,\mu_{m}$ be measures on $Y_{1},\ldots ,Y_{m}$, respectively. What is the smallest D>0 such that for all nonnegative $f_{1},\ldots ,f_{m}$ on $Y_{1},\ldots Y_{m}$ and $g_{1},\ldots ,g_{l}$ on $Z_{1},\ldots ,Z_{l}$ satisfying:

(10) $$\begin{array}{c} \prod_{i=1}^{l}g_{i}^{b_{i}}\left(\psi_{i}\left(x\right)\right)\leq \prod_{j=1}^{m}f_{j}^{c_{j}}\left(\phi_{j}\left(x\right)\right),\;\forall x, \end{array}$$

we have:

(11) $$\begin{array}{c} \prod_{i=1}^{l}{\left(\int g_{i}d\nu_{i}\right)}^{b_{i}}\leq D\prod_{j=1}^{m}{\left(\int f_{j}d\mu_{j}\right)}^{c_{j}}? \end{array}$$

Except for special case of l=1 (resp. m=1), it is generally not possible to deduce a simple expression from (10) for the optimal choice of $g_{i}$ (resp. $f_{j}$) in terms of the rest of the functions. We will refer to (11) as a forward-reverse Brascamp-Lieb inequality.

One of the motivations for considering multiple functions on both sides of (11) comes from multiuser information theory: independently, but almost simultaneously with the discovery of the Brascamp-Lieb inequality in mathematical physics, in the late 1970s, information theorists including Ahslwede, Gács and Körner [36,37] invented the image-size technique for proving strong converses in source and channel networks. An image-size inequality is a characterization of the tradeoff of the measures of certain sets connected by given random transformations (channels); we refer the interested readers to [37] for expositions on the image-size problem. Although not the way treated in [36,37], an image-size inequality can essentially be obtained from a functional inequality similar to (11) by taking the functions to be (roughly speaking) the indicator functions of sets. In the case of (10), the forward channels $\phi_{1},\ldots ,\phi_{m}$ and the reverse channels $\psi_{1},\ldots ,\psi_{l}$ degenerate into deterministic functions. In this paper, motivated by information theoretic applications similar to those of the image-size problems, we will consider further generalizations of (11) to the case of random transformations. Since the functional inequality is not restricted to indicator functions, it is strictly stronger than the corresponding image-size inequality. As a side remark, [38] uses functional inequalities that are variants of (11) together with a reverse hypercontractivity machinery to improve the image-size plus the blowing-up machinery of [39] and shows that the non-indicator function generalization is crucial for achieving the optimal scaling of the second-order rate expansion.

Of course, to justify the proposal of (11), we must also prove that (11) enjoys certain nice mathematical properties; this is the main goal of the present paper. Specifically, we focus on two aspects of (11): equivalent entropic formulation and Gaussian optimality.

In the mathematical literature, e.g., [32,36,40,41,42,43,44,45,46], it is known that certain integral inequalities are equivalent to inequalities involving relative entropies. In particular, Carlen, Loss and Lieb [47] and Carlen and Cordero-Erausquin [32] proved that the Brascamp-Lieb inequality is equivalent to the superadditivity of relative entropy. In this paper, we prove that the forward-reverse Brascamp-Lieb inequality (11) also has an entropic formulation, which turns out to be very close to the rate region of certain multiuser information theory problems (but we will clarify the difference in the text). In fact, Ahlswede, Csiszár and Körner [37,39] essentially derived image-size inequalities from similar entropic inequalities. Because of the reverse part, the proof of the equivalence of (11) and corresponding entropic inequality is more involved than the forward case considered in [32] beyond the case of finite X, $Y_{j}$, $Z_{i}$, and certain machinery from min-max theory appears necessary. In particular, the proof involves a novel use of the Legendre-Fenchel duality theory. Next, we give a basic version of our main result on the functional-entropic duality (more general versions will be given later). In order to streamline its presentation, all formal definitions of notation are postponed to Section 2.

**Theorem 1** (Dual formulation of the forward-reverse Brascamp-Lieb inequality). *Assume that:*

*(i)*  *m and l are positive integers; d∈R, X is a compact metric space;*
*(ii)*  *$b_{i}\in \left(0,\infty \right)$, $\nu_{i}$ is a finite Borel measure on a Polish space $Z_{i}$, and $Q_{Z_{i}|X}$ is a random transformation from X to $Z_{i}$, for each i=1,…,l;*
*(iii)*  *$c_{j}\in \left(0,\infty \right)$, $\mu_{j}$ is a finite Borel measure on a Polish space $Y_{j}$, and $Q_{Y_{j}|X}$ is a random transformation from X to $Y_{i}$, for each j=1,…,m;*
*(iv)*  *For any ${\left(P_{Z_{i}}\right)}_{i=1}^{l}$ such that $\sum_{i=1}^{l}D\left(P_{Z_{i}}∥\nu_{i}\right)<\infty$, there exists $P_{X}$ such that $P_{X}\to Q_{Z_{i}|X}\to P_{Z_{i}}$, i=1,…,l and $\sum_{j=1}^{m}D\left(P_{Y_{j}}∥\mu_{j}\right)<\infty$, where $P_{X}\to Q_{Y_{j}|X}\to P_{Y_{j}}$, j=1,…,m.*

*Then, the following two statements are equivalent:*

*1*. *If the nonnegative continuous functions $\left(g_{i}\right)$, $\left(f_{j}\right)$ are bounded away from zero and satisfy:*
  (12) $$\begin{array}{c} \sum_{i=1}^{l}b_{i}Q_{Z_{i}|X}\left(g_{i}\right)\leq \sum_{j=1}^{m}c_{j}Q_{Y_{j}|X}\left(f_{j}\right) \end{array}$$
  *then:*
  (13) $$\begin{array}{c} \prod_{i=1}^{l}{\left(\int g_{i}d\nu_{i}\right)}^{b_{i}}\leq \exp\left(d\right)\prod_{j=1}^{m}{\left(\int f_{j}d\mu_{j}\right)}^{c_{j}} \end{array}$$
*2*. *For any $\left(P_{Z_{i}}\right)$ such that $D(P_{Z_{i}}∥\nu_{i})<\infty$ (of course, this assumption is not essential (if we adopt the convention that the infimum in (14) is +∞ when it runs over an empty set)), i=1,…,l,*
  (14) $$\begin{array}{c} \sum_{i=1}^{l}b_{i}D\left(P_{Z_{i}}∥\nu_{i}\right)+d\geq \underset{P_{X}}{\inf}\sum_{j=1}^{m}c_{j}D\left(P_{Y_{j}}∥\mu_{j}\right) \end{array}$$
  *where $P_{X}\to Q_{Y_{j}|X}\to P_{Y_{j}}$, j=1,…,m, and the infimum is over $P_{X}$ such that $P_{X}\to Q_{Z_{i}|X}\to P_{Z_{i}}$, i=1,…,l.*

Next, in a similar vein as the proverbial result that “Gaussian functions are optimal” for the forward or the reverse Brascamp-Lieb inequality, we show in this paper that Gaussian functions are also optimal for the forward-reverse Brascamp-Lieb inequality, particularized to the case of Gaussian reference measures and linear maps. The proof scheme is based on rotational invariance (3), which can be traced back in the functional setting to Lieb [29]. More specifically, we use a variant for the entropic setting introduced by Geng and Nair [48], thereby taking advantage of the dual formulation of Theorem 1.

**Theorem** **2.** *Consider $b_{1},\ldots ,b_{l},c_{1},\ldots ,c_{m},D\in \left(0,\infty \right)$. Let $E_{1},\ldots ,E_{l},E^{1},\ldots ,E^{m}$ be Euclidean spaces, and let $B_{ji}:E_{i}\to E^{j}$ be a linear map for each i∈{1,…,l} and j∈{1,…,m}. Then, for all continuous functions $f_{j}:E^{j}\to \left[0,+\infty \right)$, $g_{i}:E_{i}\to \left[0,\infty \right)$ satisfying:*

(15) $$\begin{array}{c} \prod_{i=1}^{l}g_{i}^{b_{i}}\left(x_{i}\right)\leq \prod_{j=1}^{m}f_{j}^{c_{j}}\left(\sum_{i=1}^{l}B_{ji}x_{i}\right),\;\forall x_{1},\ldots ,x_{l}, \end{array}$$

*we have:*

(16) $$\begin{array}{c} \prod_{i=1}^{l}{\left(\int g_{i}\right)}^{b_{i}}\leq D\prod_{j=1}^{m}{\left(\int f_{j}\right)}^{c_{j}}, \end{array}$$

*if and only if for all centered Gaussian functions $f_{1},\ldots ,f_{m},g_{1},\ldots ,g_{l}$ satisfying* (15), *we have* (16).

As mentioned, in the literature on the forward or the reverse Brascamp-Lieb inequalities, it is known that a certain geometric condition (5) ensures that the best constant equals one. Now, for the forward-reverse inequality, there is a simple example where the best constant equals one:

**Example** **1.** *Let l be a positive integer, and let $M:={\left(m_{ji}\right)}_{1\leq j\leq l,1\leq i\leq l}$ be an orthogonal matrix. For any nonnegative continuous functions ${\left(f_{j}\right)}_{j=1}^{l}$${\left(g_{i}\right)}_{i=1}^{l}$ on R such that:*

(17) $$\begin{array}{c} \prod_{i=1}^{l}g_{i}\left(x_{i}\right)\leq \prod_{j=1}^{l}f_{j}\left(\sum_{i=1}^{l}m_{ji}x_{i}\right),\;\forall x^{l}\in R^{l}, \end{array}$$

*we have:*

(18) $$\begin{array}{c} \prod_{i=1}^{l}\int g_{i}\left(x\right)dx\leq \prod_{i=1}^{l}\int f_{j}\left(x\right)dx. \end{array}$$

The rest of the paper is organized as follows: Section 2 defines the notation and reviews some basic theory of convex duality. Section 3 proves Theorem 1 and also presents its extensions to the settings of noncompact spaces or general reverse channels. Section 4 proves the Gaussian optimality in the entropic formulation, with the caveat that a certain “non-degenerate” assumption is imposed to ensure the existence of extremizers. At the end of Section 4, we give a proof sketch of Example 1 and also propose a generalization of the example. To completely prove Theorem 2, in Appendix F, we use a limiting argument to drop the non-degenerate assumption and apply the equivalence between the functional and entropic formulations.

## 2. Review of the Legendre-Fenchel Duality Theory

Our proof of the equivalence of the functional and the entropic inequalities uses the Legendre-Fenchel duality theory, a topic from convex analysis. Before getting into that, a recap of some basics on the duality of topological vector spaces seems appropriate. Unless otherwise indicated, we assume Polish spaces and Borel measures. Recall that metric space. It enjoys several nice properties that we use heavily in this section, including the Prokhorov theorem and the Riesz-Kakutani theorem. Of course, the Polish space assumption covers the cases of Euclidean and discrete spaces (endowed with the Hamming metric, which induces the discrete topology, making every function on the discrete set continuous), among others. Readers interested in discrete spaces only may refer to the (much simpler) argument in [49] based on the KKT condition.

**Notation** **1.** *Let X be a topological space.*

- *$C_{c}\left(X\right)$ denotes the space of continuous functions on X with a compact support;*
- *$C_{0}\left(X\right)$ denotes the space of all continuous functions f on X that vanish at infinity (i.e., for any ϵ>0, there exists a compact set K⊆X such that |f(x)|<ϵ for x∈X\K);*
- *$C_{b}\left(X\right)$ denotes the space of bounded continuous functions on X;*
- *M(X) denotes the space of finite signed Borel measures on X;*
- *P(X) denotes the space of probability measures on X.*

We consider $C_{c}$, $C_{0}$ and $C_{b}$ as topological vector spaces, with the topology induced from the sup norm. The following theorem, usually attributed to Riesz, Markov and Kakutani, is well known in functional analysis and can be found in, e.g., [50,51].

**Theorem 3** (Riesz-Markov-Kakutani). *If X is a locally compact, σ-compact Polish space, the dual (the dual of a topological vector space consists of all continuous linear functionals on that space, which is naturally also topological vector space (with the weak${}^{*}$ topology)) of both $C_{c}\left(X\right)$ and $C_{0}\left(X\right)$ is M(X).*

**Remark** **1.** *The dual space of $C_{b}\left(X\right)$ can be strictly larger than M(X), since it also contains those linear functionals that depend on the “limit at infinity” of a function $f\in C_{b}\left(X\right)$ (originally defined for those f that do have a limit at infinity and then extended to the whole $C_{b}\left(X\right)$ by the Hahn-Banach theorem; see, e.g., [50]).*

Of course, any μ∈M(X) is a continuous linear functional on $C_{0}\left(X\right)$ or $C_{c}\left(X\right)$, given by:

(19) $$\begin{array}{c} f\mapsto \int fd\mu \end{array}$$

where *f* is a function in $C_{0}\left(X\right)$ or $C_{c}\left(X\right)$. As is well known, Theorem 3 states that the converse is also true under mild regularity assumptions on the space. Thus, we can view measures as continuous linear functionals on a certain function space (in fact, some authors prefer to construct measure theory by defining a measure as a linear functional on a suitable measure space; see Lax [50] or Bourbaki [52]); this justifies the shorthand notation:

(20) $$\begin{array}{c} \mu (f):=\int fd\mu \end{array}$$

which we employ in the rest of the paper. This viewpoint is the most natural for our setting since in the proof of the equivalent formulation of the forward-reverse Brascamp-Lieb inequality, we shall use the Hahn-Banach theorem to show the existence of certain linear functionals.

**Definition** **1.** *Let $\Lambda :C_{b}\left(X\right)\to \left(-\infty ,+\infty \right]$ be a lower semicontinuous, proper convex function. Its Legendre-Fenchel transform $\Lambda^{*}:C_{b}{\left(X\right)}^{*}\to \left(-\infty ,+\infty \right]$ is given by:*

(21) $$\begin{array}{c} \Lambda^{*}\left(\ell \right):=\underset{u\in C_{b}\left(X\right)}{\sup}\left[\ell \left(u\right)-\Lambda \left(u\right)\right]. \end{array}$$

Let ν be a nonnegative finite Borel measure on a Polish space X, and define the convex functional on $C_{b}\left(X\right)$:

(22) $$\begin{array}{cc} \Lambda (f) & :=\log\nu (\exp(f)) \end{array}$$

(23) $$\begin{array}{c} =\log\int \exp(f)d\nu . \end{array}$$

Then, note that the relative entropy has the following alternative definition: for any μ∈M(X),

(24) $$\begin{array}{c} D\left(\mu ∥\nu \right):=\underset{f\in C_{b}\left(X\right)}{\sup}\left[\mu \left(f\right)-\Lambda \left(f\right)\right] \end{array}$$

which agrees with the more familiar definition $D\left(\mu ∥\nu \right):=\mu \left(\log\frac{d\mu }{d\nu }\right)$ when ν is a probability measure, by the Donsker-Varadhan formula (cf. [53] Lemma 6.2.13). If μ is not a probability measure, then D(μ∥ν) as defined in (24) is +∞.

Given a bounded linear operator $T:C_{b}\left(Y\right)\to C_{b}\left(X\right)$, the dual operator $T^{*}:C_{b}{\left(X\right)}^{*}\to C_{b}{\left(Y\right)}^{*}$ is defined in terms of:

(25) $$\begin{array}{cc} T^{*}\mu_{X} & :C_{b}\left(Y\right)\to R;\\ f & \mapsto \mu_{X}\left(Tf\right), \end{array}$$

for any $\mu_{X}\in C_{b}{\left(X\right)}^{*}$. Since $P\left(X\right)\subseteq M\left(X\right)\subseteq C_{b}{\left(X\right)}^{*}$, *T* is said to be a conditional expectation operator if $T^{*}P\in P\left(Y\right)$ for any P∈P(X). The operator $T^{*}$ is defined as the dual of a conditional expectation operator *T* and, in a slight abuse of terminology, is said to be a random transformation from X to Y.

For example, in the notation of Theorem 1, if $g\in C_{b}\left(Y\right)$ and $Q_{Y|X}$ is a random transformation from X to Y, the quantity $Q_{Y|X}\left(g\right)$ is a function on X, defined by taking the conditional expectation. Furthermore, if $P_{X}\in P\left(X\right)$, we write $P_{X}\to Q_{Y|X}\to P_{Y}$ to indicate that $P_{Y}\in P\left(Y\right)$ is the measure induced on Y by applying $Q_{Y|X}$ to $P_{X}$.

**Figure 1:**
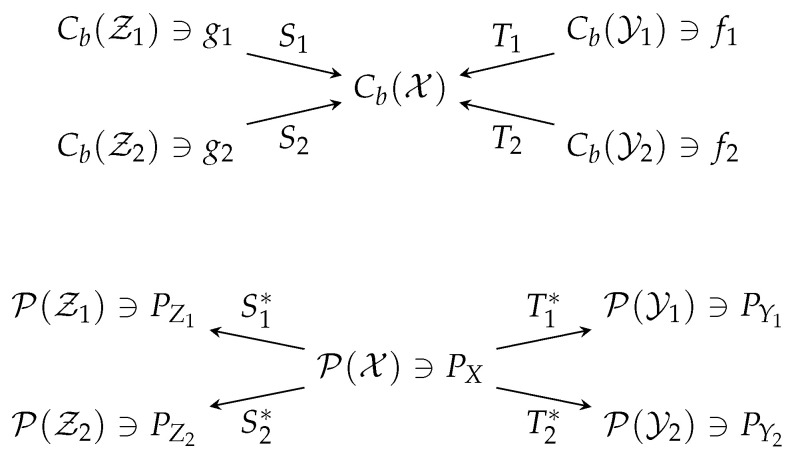
Diagrams for Theorem 1.

**Remark** **2.** *From the viewpoint of category theory (see for example [54,55]), $C_{b}$ is a functor from the category of topological spaces to the category of topological vector spaces, which is contra-variant because for any continuous, ϕ:X→Y (morphism between topological spaces), we have $C_{b}\left(\phi \right):C_{b}\left(Y\right)\to C_{b}\left(X\right)$, u↦u∘f where u∘ϕ denotes the composition of two continuous functions, reversing the arrows in the maps (i.e., the morphisms). On the other hand, M is a covariant functor and M(ϕ):M(X)→M(Y), $\mu \mapsto \mu \circ \phi^{-1}$, where $\mu \circ \phi^{-1}\left(B\right):=\mu \left(\phi^{-1}\left(B\right)\right)$ for any Borel measurable B⊆Y. “Duality” itself is a contra-variant functor between the category of topological spaces (note the reversal of arrows in Figure 1). Moreover, $C_{b}{\left(X\right)}^{*}=M\left(X\right)$ and $C_{b}{\left(\phi \right)}^{*}=M\left(\phi \right)$ if X and Y are compact metric spaces and ϕ:X→Y is continuous. Definition 2 can therefore be viewed as the special case where ϕ is the projection map:*

**Definition** **2.** *Suppose $\phi :Z_{1}\times Z_{2}\to Z_{1},\;\left(z_{1},z_{2}\right)\mapsto z_{1}$ is the projection to the first coordinate.*

- *$C_{b}\left(\phi \right):C_{b}\left(Z_{1}\right)\to C_{b}\left(Z_{1}\times Z_{2}\right)$ is called a canonical map, whose action is almost trivial: it sends a function of $z_{i}$ to itself, but viewed as a function of $\left(z_{1},z_{2}\right)$.*
- *$M\left(\phi \right):M\left(Z_{1}\times Z_{2}\right)\to M\left(Z_{1}\right)$ is called marginalization, which simply takes a joint distribution to a marginal distribution.*

The Fenchel-Rockafellar duality (see [40] Theorem 1.9, or [56] in the case of finite dimensional vector spaces) usually refers to the k=1 special case of the following result.

**Theorem** **4.** *Assume that A is a topological vector space whose dual is $A^{*}$. Let $\Theta_{j}:A\to R\cup \left\{+\infty \right\}$, j=0,1,…,k, for some positive integer k. Suppose there exist some ${\left(u_{j}\right)}_{j=1}^{k}$ and $u_{0}:=-\left(u_{1}+\ldots +u_{k}\right)$ such that:*

(26) $$\begin{array}{c} \Theta_{j}\left(u_{j}\right)<\infty ,\;j=0,\ldots ,k \end{array}$$

*and $\Theta_{0}$ is upper semicontinuous at $u_{0}$. Then:*

(27) $$\begin{array}{c} -\underset{\ell \in A^{*}}{\inf}\left[\sum_{j=0}^{k}\Theta_{j}^{*}\left(\ell \right)\right]=\underset{u_{1},\ldots ,u_{k}\in A}{\inf}\left[\Theta_{0}\left(-\sum_{j=1}^{k}u_{j}\right)+\sum_{j=1}^{k}\Theta_{j}\left(u_{j}\right)\right]. \end{array}$$

For completeness, we provide a proof of this result, which is based on the Hahn-Banach theorem (Theorem 5) and is similar to the proof of [40] Theorem 1.9.

**Proof.**  Let $m_{0}$ be the right side of (27). The ≤ part of (27) follows trivially from the (weak) min-max inequality since:

(28) $$\begin{array}{cc} m_{0} & =\underset{u_{0},\ldots ,u_{k}\in A}{\inf}\underset{\ell \in A^{*}}{\sup}\left\{\sum_{j=0}^{k}\Theta_{j}\left(u_{j}\right)-\ell \left(\sum_{j=0}^{k}u_{j}\right)\right\}\\ & \geq \underset{\ell \in A^{*}}{\sup}\underset{u_{0},\ldots ,u_{k}\in A}{\inf}\left\{\sum_{j=0}^{k}\Theta_{j}\left(u_{j}\right)-\ell \left(\sum_{j=0}^{k}u_{j}\right)\right\}\\ & =-\underset{\ell \in A^{*}}{\inf}\left[\sum_{j=0}^{k}\Theta_{j}^{*}\left(\ell \right)\right]. \end{array}$$

It remains to prove the ≥ part, and it suffices to assume without loss of generality that $m_{0}>-\infty$. Note that (26) also implies that $m_{0}<+\infty$. Define convex sets:

(29) $$\begin{array}{cc} C_{j} & :=\{\left(u,r\right)\in A\times R:\;r>\Theta_{j}\left(u\right)\},\;j=0,\ldots ,k; \end{array}$$

(30) $$\begin{array}{cc} B & :=\{\left(0,m\right)\in A\times R:\;m\leq m_{0}\}. \end{array}$$

Observe that these are nonempty sets because of (26). Furthermore, $C_{0}$ has a nonempty interior by the assumption that $\Theta_{0}$ is upper semicontinuous at $u_{0}$. Thus, the Minkowski sum:

(31) $$\begin{array}{c} C:=C_{0}+\ldots +C_{k} \end{array}$$

is a convex set with a nonempty interior. Moreover, C∪B=∅. By the Hahn-Banach theorem (Theorem 5), there exists $\left(\ell ,s\right)\in A^{*}\times R$ such that:

(32) $$\begin{array}{c} sm\leq \ell \left(\sum_{j=0}^{k}u_{j}\right)+s\sum_{j=0}^{k}r_{j}. \end{array}$$

For any $m\leq m_{0}$ and $\left(u_{j},r_{j}\right)\in C_{j}$, j=0,…,k. From (30), we see (32) can only hold when s≥0. Moreover, from (26) and the upper semicontinuity of $\Theta_{0}$ at $u_{0}$, we see that the $\sum_{j=0}^{k}u_{j}$ in (32) can take a value in a neighborhood of 0∈A; hence, s≠0. Thus, by dividing *s* on both sides of (32) and setting ℓ←−ℓ/s, we see that:

(33) $$\begin{array}{cc} m_{0} & \leq \underset{u_{0},\ldots ,u_{k}\in A}{\inf}\left[-\ell \left(\sum_{j=0}^{k}u_{j}\right)+\sum_{j=0}^{k}\Theta_{j}\left(u_{j}\right)\right]\\ & =-\left[\sum_{j=0}^{k}\Theta_{j}^{*}\left(\ell \right)\right] \end{array}$$

which establishes ≥ in (27). ☐

**Theorem 5** (Hahn-Banach) *Let C and B be convex, nonempty disjoint subsets of a topological vector space A.*

*1*. *If the interior of C is non-empty, then there exists $\ell \in A^{*}$, ℓ≠0 such that:*
  (34) $$\begin{array}{c} \underset{u\in B}{\sup}\ell \left(u\right)\leq \underset{u\in C}{\inf}\ell \left(u\right). \end{array}$$
*2*. *If A is locally convex, B is compact and C is closed, then there exists $\ell \in A^{*}$ such that:*
  (35) $$\begin{array}{c} \underset{u\in B}{\sup}\ell \left(u\right)<\underset{u\in C}{\inf}\ell \left(u\right). \end{array}$$

**Remark** **3.** *The assumption in Theorem 5 that C has a nonempty interior is only necessary in the infinite dimensional case. However, even if A in Theorem 4 is finite dimensional, the assumption in Theorem 4 that $\Theta_{0}$ is upper semicontinuous at $u_{0}$ is still necessary, because this assumption was not only used in applying Hahn-Banach, but also in concluding that s≠0 in* (32).

## 3. The Entropic-Functional Duality

In this section, we prove Theorem 1 and some of its generalizations.

### 3.1. Compact X

We first state a duality theorem for the case of compact spaces to streamline the proof. Later, we show that the argument can be extended to a particular non-compact case (Theorem 1 is not included in the conference paper [49], but was announced in the conference presentation). Our proof based on the Legendre-Fenchel duality (Theorem 4) was inspired by the proof of the Kantorovich duality in the theory of optimal transportation (see [40] Chapter 1, where the idea was credited to Brenier).

Recall from Section 2 that a random transformation (a mapping between probability measures) is formally the dual of a conditional expectation operator. Suppose $P_{Y_{j}|X}=T_{j}^{*}$, j=1,…,m and $P_{Z_{i}|X}=S_{i}^{*}$, i=1,…,l.

**Proof of Theorem 1.**  We can safely assume d=0 below without loss of generality (since otherwise, we can always substitute $\mu_{1}\leftarrow \exp\left(\frac{d}{c_{1}}\right)\mu_{1}$).

**1)⇒2)**  This is the nontrivial direction, which relies on certain (strong) min-max type results. In Theorem 4, put (in (36), u≤0 means that *u* is pointwise non-positive):
  (36) $$\begin{array}{c} \Theta_{0}:u\in C_{b}\left(X\right)\mapsto \left\{\begin{array}{cc} 0 & u\leq 0;\\ +\infty & \mathrm{otherwise}. \end{array}\right. \end{array}$$
  Then,
  (37) $$\begin{array}{c} \Theta_{0}^{*}:\pi \in M\left(X\right)\mapsto \left\{\begin{array}{cc} 0 & \pi \geq 0;\\ +\infty & \mathrm{otherwise}. \end{array}\right. \end{array}$$
  For each j=1,…,m, set:
  (38) $$\begin{array}{c} \Theta_{j}\left(u\right):=c_{j}\inf\log\mu_{j}\left(\exp\left(\frac{1}{c_{j}}v\right)\right) \end{array}$$
  where the infimum is over $v\in C_{b}\left(Y\right)$ such that $u=T_{j}v$; if there is no such *v*, then $\Theta_{j}\left(u\right):=+\infty$ as a convention. Observe that:
  - $\Theta_{j}$ is convex: indeed, given arbitrary $u^{0}$ and $u^{1}$, suppose that $v^{0}$ and $v^{1}$ respectively achieve the infimum in (38) for $u^{0}$ and $u^{1}$ (if the infimum is not achievable, the argument still goes through by the approximation and limit argument). Then, for any α∈[0,1], $v^{\alpha }:=\left(1-\alpha \right)v^{0}+\alpha v^{1}$ satisfies $u^{\alpha }=T_{j}v^{\alpha }$ where $u^{\alpha }:=\left(1-\alpha \right)u^{0}+\alpha u^{1}$. Thus, the convexity of $\Theta_{j}$ follows from the convexity of the functional in (23);
  - $\Theta_{j}\left(u\right)>-\infty$ for any $u\in C_{b}\left(X\right)$. Otherwise, for any $P_{X}$ and $P_{Y_{j}}:=T_{j}^{*}P_{X}$, we have:
    (39) $$\begin{array}{cc} D(P_{Y_{j}}∥\mu_{j}) & =\underset{v}{\sup}\left\{P_{Y_{j}}\left(v\right)-\log\mu_{j}\left(\exp\left(v\right)\right)\right\} \end{array}$$
    (40) $$\begin{array}{c} =\underset{v}{\sup}\left\{P_{X}\left(T_{j}v\right)-\log\mu_{j}\left(\exp\left(v\right)\right)\right\} \end{array}$$
    (41) $$\begin{array}{c} =\underset{u\in C_{b}\left(X\right)}{\sup}\left\{P_{X}\left(u\right)-\frac{1}{c_{j}}\Theta_{j}\left(c_{j}u\right)\right\} \end{array}$$
    (42) $$\begin{array}{c} =+\infty \end{array}$$
    which contradicts the assumption that $\sum_{j=1}^{m}c_{j}D\left(P_{Y_{j}}∥\mu_{j}\right)<\infty$ in the theorem;
  - From Steps (39)–(41), we see $\Theta_{j}^{*}\left(\pi \right)=c_{j}D\left(T_{j}^{*}\pi ∥\mu_{j}\right)$ for any π∈M(X), where the definition of $D(\cdot ∥\mu_{j})$ is extended using the Donsker-Varadhan formula (that is, it is infinite when the argument is not a probability measure).
  Finally, for the given ${\left(P_{Z_{i}}\right)}_{i=1}^{l}$, choose:
  (43) $$\begin{array}{c} \Theta_{m+1}:u\in C_{b}\left(X\right)\mapsto \left\{\begin{array}{cc} \sum_{i=1}^{l}P_{Z_{i}}\left(w_{i}\right) & \mathrm{if}u=\sum_{i=1}^{l}S_{i}w_{i}\;\mathrm{for}\;\mathrm{some}\;w_{i}\in C_{b}\left(Z_{i}\right);\\ +\infty & \mathrm{otherwise}. \end{array}\right. \end{array}$$
  Notice that:
  - $\Theta_{m+1}$ is convex;
  - $\Theta_{m+1}$ is well defined (that is, the choice of $\left(w_{i}\right)$ in (43) is inconsequential). Indeed, if ${\left(w_{i}\right)}_{i=1}^{l}$ is such that $\sum_{i=1}^{l}S_{i}w_{i}=0$, then:
    (44) $$\begin{array}{cc} \sum_{i=1}^{l}P_{Z_{i}}\left(w_{i}\right) & =\sum_{i=1}^{l}S_{i}^{*}P_{X}\left(w_{i}\right)\\ & =\sum_{i=1}^{l}P_{X}\left(S_{i}w_{i}\right)\\ & =0, \end{array}$$
    where $P_{X}$ is such that $S_{i}^{*}P_{X}=P_{Z_{i}}$, i=1,…,l, whose existence is guaranteed by the assumption of the theorem. This also shows that $\Theta_{m+1}>-\infty$.
  - (45) $$\begin{array}{cc} \Theta_{m+1}^{*}\left(\pi \right) & :=\underset{u}{\sup}\left\{\pi \left(u\right)-\Theta_{m+1}\left(u\right)\right\}\\ & =\underset{w_{1},\ldots ,w_{l}}{\sup}\left\{\pi \left(\sum_{i=1}^{l}S_{i}w_{i}\right)-\sum_{i=1}^{l}P_{Z_{i}}\left(w_{i}\right)\right\}\\ & =\underset{w_{1},\ldots ,w_{l}}{\sup}\left\{\sum_{i=1}^{l}S_{i}^{*}\pi \left(w_{i}\right)-\sum_{i=1}^{l}P_{Z_{i}}\left(w_{i}\right)\right\}\\ & =\left\{\begin{array}{cc} 0 & \mathrm{if}S_{i}^{*}\pi =P_{Z_{i}},\;i=1,\ldots ,l;\\ +\infty & \mathrm{otherwise}. \end{array}\right. \end{array}$$
  Invoking Theorem 4 (where the $u_{j}$ in Theorem 4 can be chosen as the constant function $u_{j}\equiv 1$, j=1,…,m+1):
  (46) $$\begin{array}{c} \;\underset{\pi :\pi \geq 0,\;S_{i}^{*}\pi =P_{Z_{i}}}{\inf}\sum_{j=1}^{m}c_{j}D\left(T_{j}^{*}\pi ∥\mu_{j}\right)\\ =-\underset{v^{m},w^{l}:\sum_{j=1}^{m}T_{j}v_{j}+\sum_{i=1}^{l}S_{i}w_{i}\geq 0}{\inf}\left[\sum_{j=1}^{m}c_{j}\log\mu_{j}\left(\exp\left(\frac{1}{c_{j}}v_{j}\right)\right)+\sum_{i=1}^{l}P_{Z_{i}}\left(w_{i}\right)\right] \end{array}$$
  where $v^{m}$ denotes the collection of the functions $v_{1},\ldots ,v_{m}$, and similarly for $w^{l}$. Note that the left side of (46) is exactly the right side of (14). For any ϵ>0, choose $v_{j}\in C_{b}\left(Y_{j}\right)$, j=1,…,m and $w_{i}\in C_{b}\left(Z_{i}\right)$, i=1,…,l such that $\sum_{j=1}^{m}T_{j}v_{j}+\sum_{i=1}^{l}S_{i}w_{i}\geq 0$ and:
  (47) $$\begin{array}{c} \epsilon -\sum_{j=1}^{m}c_{j}\log\mu_{j}\left(\exp\left(\frac{1}{c_{j}}v_{j}\right)\right)-\sum_{i=1}^{l}P_{Z_{i}}\left(w_{i}\right)>\underset{\pi :\pi \geq 0,\;S_{i}^{*}\pi =P_{Z_{i}}}{\inf}\sum_{j=1}^{m}c_{j}D\left(T_{j}^{*}\pi ∥\mu_{j}\right) \end{array}$$
  Now, invoking (13) with $f_{j}:=\exp\left(\frac{1}{c_{j}}v_{j}\right)$, j=1,…,m and $g_{i}:=\exp\left(-\frac{1}{b_{i}}w_{i}\right)$, i=1,…,l, we upper bound the left side of (47) by:
  (48) $$\begin{array}{c} \epsilon -\sum_{i=1}^{l}b_{i}\log\nu_{i}\left(g_{i}\right)+\sum_{i=1}^{l}b_{i}P_{Z_{i}}\left(\log g_{i}\right)\leq \epsilon +\sum_{i=1}^{l}b_{i}D\left(P_{Z_{i}}∥\nu_{i}\right) \end{array}$$
  where the last step follows by the Donsker-Varadhan formula. Therefore, (14) is established since ϵ>0 is arbitrary.
**2)⇒1)**  Since $\nu_{i}$ is finite and $g_{i}$ is bounded by assumption, we have $\nu_{i}\left(g_{i}\right)<\infty$, i=1,…,l. Moreover, (13) is trivially true when $\nu_{i}\left(g_{i}\right)=0$ for some *i*, so we will assume below that $\nu_{i}\left(g_{i}\right)\in \left(0,\infty \right)$ for each *i*. Define $P_{Z_{i}}$ by:
  (49) $$\begin{array}{c} \frac{dP_{Z_{i}}}{d\nu_{i}}=\frac{g_{i}}{\nu_{i}\left(g_{i}\right)},\;i=1,\ldots ,l. \end{array}$$
  Then, for any ϵ>0,
  (50) $$\begin{array}{cc} \sum_{i=1}^{l}b_{i}\log\nu_{i}\left(g_{i}\right) & =\sum_{i=1}^{l}b_{i}\left[P_{Z_{i}}\left(\log g_{i}\right)-D\left(P_{Z_{i}}∥\nu_{i}\right)\right] \end{array}$$
  (51) $$\begin{array}{cc} \; & <\sum_{j=1}^{m}c_{j}P_{Y_{j}}\left(\log f_{j}\right)+\epsilon -\sum_{j=1}^{m}c_{j}D\left(P_{Y_{j}}∥\mu_{j}\right) \end{array}$$
  (52) $$\begin{array}{cc} \; & \leq \epsilon +\sum_{j=1}^{m}c_{j}\log\mu_{j}\left(f_{j}\right) \end{array}$$
  where:
  - (51) uses the Donsker-Varadhan formula, and we have chosen $P_{X}$, $P_{Y_{j}}:=T_{j}^{*}P_{X}$, j=1,…,m such that:
    (53) $$\begin{array}{c} \sum_{i=1}^{l}b_{i}D\left(P_{Z_{i}}∥\nu_{i}\right)>\sum_{j=1}^{m}c_{j}D\left(P_{Y_{j}}∥\mu_{j}\right)-\epsilon \end{array}$$
  - (52) also follows from the Donsker-Varadhan formula.
  The result follows since ϵ>0 can be arbitrary.
  ☐

**Remark** **4.** *Condition (iv) in the theorem imposes a rather strong assumption on $\left(S_{i}\right)$: for simplicity, consider the case where $\left|X|,\right|Z_{i}|<\infty$. Then, Condition (iv) assumes that for any $\left(P_{Z_{i}}\right)$, there exists $P_{X}$ such that $P_{Z_{i}}=S_{i}^{*}P_{X}$. This assumption is certainly satisfied when $\left(S_{i}\right)$ are induced by coordinate projections; the case of l=1 and $P_{Z|X}$ being a reverse erasure channel gives a simple example where $P_{Z|X}$ is not a deterministic map.*

Next, we give a generalization of Theorem 1, which alleviates the restriction on $\left(S_{i}\right)$:

**Theorem** **6.** *Theorem 1 continues to hold if Condition (iv) therein is weakened to the following:*

- *For any $P_{X}$ such that $D(S_{i}^{*}P_{X}∥\nu_{i})<\infty$, i=1,…,l, there exists ${\tilde{P}}_{X}$ such that $S_{i}^{*}{\tilde{P}}_{X}=S_{i}^{*}P_{X}$ for each i and $\sum_{j=1}^{m}c_{j}D\left(T_{j}^{*}{\tilde{P}}_{X}∥\mu_{j}\right)<\infty$ for each j.*

*and the conclusion of the theorem will be replaced by the equivalence of the following two statements:*

*1*. *For any nonnegative continuous functions $\left(g_{i}\right)$, $\left(f_{j}\right)$ bounded away from zero and such that:*
  (54) $$\begin{array}{c} \sum_{i=1}^{l}b_{i}S_{i}\log g_{i}\leq \sum_{j=1}^{m}c_{j}T_{j}\log f_{j} \end{array}$$
  *we have:*
  (55) $$\begin{array}{c} \underset{\left({\tilde{g}}_{i}\right):\sum_{i=1}^{l}b_{i}S_{i}\log{\tilde{g}}_{i}\geq \sum_{i=1}^{l}b_{i}S_{i}\log g_{i}}{\inf}\prod_{i=1}^{l}\nu_{i}^{b_{i}}\left({\tilde{g}}_{i}\right)\leq \exp\left(d\right)\prod_{j=1}^{m}\mu_{j}^{c_{j}}\left(f_{j}\right). \end{array}$$
*2*. *For any $\left(P_{X}\right)$ such that $D(S_{i}^{*}P_{X}∥\nu_{i})<\infty$, i=1,…,l,*
  (56) $$\begin{array}{c} \sum_{i=1}^{l}b_{i}D\left(S_{i}^{*}P_{X}∥\nu_{i}\right)+d\geq \underset{{\tilde{P}}_{X}:S_{i}^{*}{\tilde{P}}_{X}=S_{i}^{*}P_{X}}{\inf}\sum_{j=1}^{m}c_{j}D\left(T_{j}^{*}{\tilde{P}}_{X}∥\mu_{j}\right). \end{array}$$

In Appendix A, we show that Theorem 6 indeed recovers Theorem 1 for the more restricted class of random transformations.

**Proof.**  Here, we mention the parts of the proof that need to be changed: upon specifying $\left(f_{j}\right)$ and $\left(g_{i}\right)$ right after (47), we select $\left({\tilde{g}}_{i}\right)$ such that:

(57) $$\begin{array}{cc} \sum_{i=1}^{l}b_{i}S_{i}\log{\tilde{g}}_{i} & \geq \sum_{i=1}^{l}b_{i}S_{i}\log g_{i} \end{array}$$

(58) $$\begin{array}{cc} \sum_{i=1}^{l}b_{i}\log\nu_{i}\left({\tilde{g}}_{i}\right) & \leq \sum_{j=1}^{m}c_{j}\log\mu_{j}\left(f_{j}\right)+\epsilon . \end{array}$$

Then, in lieu of (59), we upper-bound the left side of (47) by:

(59) $$\begin{array}{c} 2\epsilon -\sum_{i=1}^{l}b_{i}\log\nu_{i}\left({\tilde{g}}_{i}\right)+\sum_{i=1}^{l}b_{i}P_{Z_{i}}\left(\log{\tilde{g}}_{i}\right)\leq 2\epsilon +\sum_{i=1}^{l}b_{i}D\left(P_{Z_{i}}∥\nu_{i}\right) \end{array}$$

which establishes the 1)⇒2) part. For the other direction, for each i∈{1,2,…,l}, define:

(60) $$\begin{array}{c} \Lambda_{i}\left(u\right):=\underset{{\tilde{g}}_{i}>0:b_{i}S_{i}\log{\tilde{g}}_{i}=u}{\inf}b_{i}\log\nu_{i}\left({\tilde{g}}_{i}\right). \end{array}$$

Then, following essentially the same proof as that of $\Theta_{j}$ in (38), we see that $\Lambda_{i}$ is proper convex and:

(61) $$\begin{array}{c} \Lambda_{i}^{*}\left(\pi \right)=b_{i}D\left(S_{i}^{*}\pi ∥\mu_{j}\right). \end{array}$$

Moreover, let:

(62) $$\begin{array}{c} \Lambda_{l+1}\left(u\right):=\left\{\begin{array}{cc} 0 & \mathrm{if}u=-\sum b_{i}S_{i}\log g_{i};\\ +\infty & \mathrm{otherwise}. \end{array}\right. \end{array}$$

Then, $\Lambda_{l+1}^{*}\left(\pi \right)=-\sum b_{i}S_{i}^{*}\pi \left(\log g_{i}\right)$. Using the Legendre-Fenchel duality, we see that for any ϵ>0,

$$\begin{array}{c} \;\underset{\left({\tilde{g}}_{i}\right):\sum_{i=1}^{l}b_{i}S_{i}\log{\tilde{g}}_{i}\geq \sum_{i=1}^{l}b_{i}S_{i}\log g_{i}}{\inf}\sum_{i=1}^{l}b_{i}\log\nu_{i}\left({\tilde{g}}_{i}\right) \end{array}$$

(63) $$\begin{array}{c} =\underset{u_{1},\ldots ,u_{l+1}}{\inf}\left\{\Theta_{0}\left(-\sum_{i=1}^{l+1}u_{i}\right)+\sum_{i=1}^{l+1}\Lambda_{i}\left(u_{i}\right)\right\} \end{array}$$

(64) $$\begin{array}{c} =\underset{\pi }{\sup}\left\{-\sum_{i=0}^{l+1}\Theta_{i}^{*}\left(\pi \right)\right\} \end{array}$$

(65) $$\begin{array}{c} =\underset{\pi \geq 0}{\sup}\left\{-\sum_{i=1}^{l+1}\Theta_{i}^{*}\left(\pi \right)\right\} \end{array}$$

(66) $$\begin{array}{c} =\underset{\pi \geq 0}{\sup}\left\{\sum_{i=1}^{l}b_{i}S_{i}^{*}\pi \left(\log g_{i}\right)-\sum_{i=1}^{l}b_{i}D\left(S_{i}^{*}\pi ∥\nu_{i}\right)\right\} \end{array}$$

(67) $$\begin{array}{c} \leq \sum_{i=1}^{l}b_{i}S_{i}^{*}P_{X}\left(\log g_{i}\right)-\sum_{i=1}^{l}b_{i}D\left(S_{i}^{*}P_{X}∥\nu_{i}\right)+\epsilon \end{array}$$

(68) $$\begin{array}{c} \leq \sum_{j=1}^{m}c_{j}T_{j}^{*}{\tilde{P}}_{X}\left(\log f_{j}\right)-\sum_{j=1}^{m}c_{j}D\left(T_{j}^{*}{\tilde{P}}_{X}∥\mu_{j}\right)+2\epsilon \end{array}$$

(69) $$\begin{array}{c} \leq 2\epsilon +\sum_{j=1}^{m}c_{j}\log\mu_{j}\left(f_{j}\right) \end{array}$$

where:

- To see (67), we note that the sup in (66) can be restricted to π, which is a probability measure, since otherwise, the relative entropy terms in (66) are +∞ by its definition via the Donsker-Varadhan formula. Then, we select $P_{X}$ such that (67) holds.
- In (68), we have chosen ${\tilde{P}}_{X}$ such that:
  (70) $$\begin{array}{cc} S_{i}^{*}{\tilde{P}}_{X} & =S_{i}^{*}P_{X},\;1\leq i\leq l; \end{array}$$
  (71) $$\begin{array}{cc} \sum_{i=1}^{l}b_{i}D\left(S_{i}^{*}P_{X}\right) & >\sum_{j=1}^{m}c_{j}D\left(T_{j}^{*}{\tilde{P}}_{X}∥\mu_{j}\right)-\epsilon , \end{array}$$
  and then applied the assumption (54). The result follows since ϵ>0 can be arbitrary.
  ☐

**Remark** **5.** *The infimum in (14) is in fact achievable: for any $\left(P_{Z_{i}}\right)$, there exists a $P_{X}$ that minimizes $\sum_{j=1}^{m}c_{j}D\left(P_{Y_{j}}∥\mu_{j}\right)$ subject to the constraints $S_{i}^{*}P_{X}=P_{Z_{i}}$, i=1,…m, where $P_{Y_{j}}:=T_{j}^{*}P_{X}$, j=1,…,m. Indeed, since the singleton $\left\{P_{Z_{i}}\right\}$ is weak${}^{*}$-closed and $S_{i}^{*}$ is weak${}^{*}$-continuous (Generally, if T:A→B is a continuous map between two topologically vector spaces, then $T^{*}:B^{*}\to A^{*}$ is a weak${}^{*}$ continuous map between the dual spaces. Indeed, if $y_{n}\to y$ is a weak${}^{*}$-convergent subsequence in $B^{*}$, meaning $y_{n}\left(b\right)\to y\left(b\right)$ for any b∈B, then, we must have $T^{*}y_{n}\left(a\right)=y_{n}\left(Ta\right)\to y\left(Ta\right)=T^{*}y\left(a\right)$ for any a∈A, meaning that $T^{*}y_{n}$ converges to $T^{*}y$ in the weak${}^{*}$ topology.), the set ${⋂}_{i=1}^{l}{\left(S_{i}^{*}\right)}^{-1}P_{Z_{i}}$ is weak${}^{*}$-closed in M(X); hence, its intersection with P(X) is weak${}^{*}$-compact in P(X), because P(X) is weak${}^{*}$-compact by (a simple version for the setting of a compact underlying space X of) the Prokhorov theorem [57]. Moreover, by the weak${}^{*}$-lower semicontinuity of $D(\cdot ∥\mu_{j})$ (easily seen from the variational formula/Donsker-Varadhan formula of the relative entropy, cf. [58]) and the weak${}^{*}$-continuity of $T_{j}^{*}$, j=1,…,m, we see that $\sum_{j=1}^{m}c_{j}D\left(T_{j}^{*}P_{X}∥\mu_{j}\right)$ is weak${}^{*}$-lower semicontinuous in $P_{X}$, and hence, the existence of a minimizing $P_{X}$ is established.*

**Remark** **6.** *Abusing the terminology from min-max theory, Theorem 1 may be interpreted as a “strong duality” result, which establishes the equivalence of two optimization problems. The 1)⇒2) part is the non-trivial direction, which requires regularity on the spaces. In contrast, the 2)⇒1) direction can be thought of as a “weak duality”, which establishes only a partial relation, but holds for more general spaces.*

### 3.2. Noncompact X

Our proof of 1)⇒2) in Theorem 1 makes use of the Hahn-Banach theorem and hence relies crucially on the fact that the measure space is the dual of the function space. Naively, one might want to extend the the proof to the case of locally compact X by considering $C_{0}\left(X\right)$ instead of $C_{b}\left(X\right)$, so that the dual space is still M(X). However, this would not work: consider the case when $X=Z_{1}\times ,\ldots ,\times Z_{l}$ and each $S_{i}$ is the canonical map. Then, $\Theta_{m+1}\left(u\right)$ as defined in (43) is +∞ unless u≡0 (because $u\in C_{0}\left(X\right)$ requires that *u* vanishes at infinity); thus, $\Theta_{m+1}^{*}\equiv 0$. Luckily, we can still work with $C_{b}\left(X\right)$; in this case, $\ell \in C_{b}{\left(X\right)}^{*}$ may not be a measure, but we can decompose it into ℓ=π+R where π∈M(X) and *R* is a linear functional “supported at infinity”. Below, we use the techniques in [40] (Chapter 1.3) to prove a particular extension of Theorem 1 to a non-compact case.

**Theorem** **7.** *Theorem 1 still holds if*

- *The assumption that X is a compact metric space is relaxed to the assumption that it is a locally compact and σ-compact Polish space;*
- *$X=\prod_{i=1}^{l}Z_{i}$ and $S_{i}:C_{b}\left(Z_{i}\right)\to C_{b}\left(X\right)$, i=1,…,l are canonical maps (see Definition 2).*

**Proof.**  The proof of the “weak duality” part 2)⇒1) still works in the noncompact case, so we only need to explain what changes need to be made in the proof of the 1)⇒2) part. Let $\Theta_{0}$ be defined as before, in (36). Then, for any $\ell \in C_{b}{\left(X\right)}^{*}$,

(72) $$\begin{array}{c} \Theta_{0}^{*}\left(\ell \right)=\underset{u\leq 0}{\sup}\ell \left(u\right) \end{array}$$

which is zero if *ℓ* is nonnegative (in the sense that ℓ(u)≥0 for every u≥0), and +∞ otherwise. This means that when computing the infimum on the left side of (27), we only need to take into account those nonnegative *ℓ*.

Next, let $\Theta_{m+1}$ be also defined as before. Then, directly from the definition, we have:

(73) $$\begin{array}{cc} \Theta_{m+1}^{*}\left(\ell \right) & =\left\{\begin{array}{cc} 0 & \mathrm{if}\ell \left(\sum_{i}S_{i}w_{i}\right)=\sum_{i}P_{Z_{i}}\left(w_{i}\right),\;\forall w_{i}\in C_{b}\left(Z_{i}\right),\;i=1,\ldots l;\\ +\infty & \mathrm{otherwise}. \end{array}\right. \end{array}$$

For any $\ell \in C_{b}^{*}\left(X\right)$. Generally, the condition in the first line of (73) does not imply that *ℓ* is a measure. However, if *ℓ* is also nonnegative, then using a technical result in [40] Lemma 1.25, we can further simplify:

(74) $$\begin{array}{cc} \Theta_{m+1}^{*}\left(\ell \right) & =\left\{\begin{array}{cc} 0 & \mathrm{if}\ell \in M\left(X\right)\mathrm{and}S_{i}^{*}\ell =P_{Z_{i}},\;i=1,\ldots ,l;\\ +\infty & \mathrm{otherwise}. \end{array}\right. \end{array}$$

This further shows that when we compute the left side of (27), the infimum can be taken over *ℓ*, which is a coupling of $\left(P_{Z_{i}}\right)$. In particular, if *ℓ* is a probability measure, then $\Theta_{j}^{*}\left(\ell \right)=c_{j}D\left(T_{j}^{*}\ell ∥\mu_{j}\right)$ still holds with the $\Theta_{j}$ defined in (38), j=1,…,m. Thus, the rest of the proof can proceed as before. ☐

**Remark** **7.** *The second assumption is made in order to achieve (74) in the proof.*

## 4. Gaussian Optimality

Recall that the conventional Brascamp-Lieb inequality and its reverse ((1) and (2)) state that centered Gaussian functions exhaust such inequalities, and in particular, verifying those inequalities is reduced to a finite dimensional optimization problem (only the covariance matrices in these Gaussian functions are to be optimized). In this section, we show that similar results hold for the forward-reverse Brascamp-Lieb inequality, as well. Our proof uses the rotational invariance argument mentioned in Section 1. Since the forward-reverse Brascamp-Lieb inequality has dual representations (Theorem 7), in principle, the rotational invariance argument can be applied either to the functional representation (as in Lieb’s paper [29]) or the entropic representation (as in Geng-Nair [48]). Here, we adopt the latter approach. We first consider a certain “non-degenerate” case where the existence of an extremizer is guaranteed. Then, Gaussian optimality in the general case follows by a limiting argument (Appendix F), establishing Theorem 2.

### 4.1. Non-Degenerate Forward Channels

This subsection focuses on the following case:

**Assumption** **1.**

- *Fix Lebesgue measures ${\left(\mu_{j}\right)}_{j=1}^{m}$ and Gaussian measures ${\left(\nu_{i}\right)}_{i=1}^{l}$ on R;*
- *non-degenerate (Definition 3 below) linear Gaussian random transformation ${\left(P_{Y_{j}|X}\right)}_{j=1}^{m}$ (where $X:=(X_{1},\ldots ,X_{l})$) associated with conditional expectation operators ${\left(T_{j}\right)}_{j=1}^{m}$;*
- *${\left(S_{i}\right)}_{i=1}^{l}$ are induced by coordinate projections;*
- *positive $\left(c_{j}\right)$ and $\left(b_{i}\right)$.*

**Definition** **3.** *We say $\left(Q_{Y_{1}|X},\ldots ,Q_{Y_{m}|X}\right)$ is non-degenerate if each $Q_{Y_{j}|X=0}$ is an $n_{j}$-dimensional Gaussian distribution with an invertible covariance matrix.*

Given Borel measures $P_{X_{i}}$ on R, i=1,…,l, define:

(75) $$\begin{array}{c} F_{0}\left(\left(P_{X_{i}}\right)\right):=\underset{P_{X}}{\inf}\sum_{j=1}^{m}c_{j}D\left(P_{Y_{j}}∥\mu_{j}\right)-\sum_{i=1}^{l}b_{i}D\left(P_{X_{i}}∥\nu_{i}\right) \end{array}$$

where the infimum is over Borel measures $P_{X}$ that have $\left(P_{X_{i}}\right)$ as marginals. Note that (75) is well defined since the first term cannot be +∞ under the non-degenerate assumption, and the second term cannot be −∞. The aim of this subsection is to prove the following:

**Theorem** **8.** $\sup_{\left(P_{X_{i}}\right)}F_{0}\left(\left(P_{X_{i}}\right)\right)$ *, where the supremum is over Borel measures $P_{X_{i}}$ on R, and i=1,…,l, is achieved by some Gaussian ${\left(P_{X_{i}}\right)}_{i=1}^{l}$, in which case the infimum in (75) is achieved by some Gaussian $P_{X}$.*

Naturally, one would expect that Gaussian optimality can be established when ${\left(\mu_{j}\right)}_{j=1}^{m}$ and ${\left(\nu_{i}\right)}_{i=1}^{l}$ are either Gaussian or Lebesgue. We made the assumption that the former is Lebesgue and the latter is Gaussian so that certain technical conditions can be justified more easily. More precisely, the following observation shows that we can regularize the distributions by a second moment constraint for free:

**Proposition** **1.** *$\sup_{\left(P_{X_{i}}\right)}F_{0}\left(\left(P_{X_{i}}\right)\right)$ is finite and there exist $\sigma_{i}^{2}\in \left(0,\infty \right)$, i=1,…,l such that it equals:*

(76) $$\begin{array}{c} \underset{\left(P_{X_{i}}\right):E\left[X_{i}^{2}\right]\leq \sigma_{i}^{2}}{\sup}F_{0}\left(\left(P_{X_{i}}\right)\right). \end{array}$$

**Proof.**  When $\mu_{j}$ is Lebesgue and $P_{Y_{j}|X}$ is non-degenerate, $D\left(P_{Y_{j}}∥\mu_{j}\right)=-h\left(P_{Y_{j}}\right)\leq -h\left(P_{Y_{j}}|X\right)$ is bounded above (in terms of the variance of the additive noise of $P_{Y_{j}|X}$). Moreover, $D(P_{X_{i}}∥\nu_{i})\geq 0$ when $\nu_{i}$ is Gaussian, so $\sup_{\left(P_{X_{i}}\right)}F_{0}\left(\left(P_{X_{i}}\right)\right)<\infty$. Further, choosing $\left(P_{X_{i}}\right)=\left(\nu_{i}\right)$ and using the covariance matrix to lower bound the first term in (75) show that $\sup_{\left(P_{X_{i}}\right)}F_{0}\left(\left(P_{X_{i}}\right)\right)>-\infty$.

To see (76), notice that:

(77) $$\begin{array}{cc} D(P_{X_{i}}∥\nu_{i}) & =D\left(P_{X_{i}}∥\nu_{i}^{\prime }\right)+E\left[{ı}_{\nu_{i}^{\prime }∥\nu_{i}}\left(X\right)\right]\\ & =D\left(P_{X_{i}}∥\nu_{i}^{\prime }\right)+D\left(\nu_{i}^{\prime }∥\nu_{i}\right)\\ & \geq D(\nu_{i}^{\prime }∥\nu_{i}) \end{array}$$

where $\nu_{i}^{\prime }$ is a Gaussian distribution with the same first and second moments as $X_{i}∼P_{X_{i}}$. Thus, $D(P_{X_{i}}∥\nu_{i})$ is bounded below by some function of the second moment of $X_{i}$, which tends to ∞ as the second moment of $X_{i}$ tends to ∞. Moreover, as argued in the preceding paragraph, the first term in (75) is bounded above by some constant depending only on $\left(P_{Y_{j}|X}\right)$. Thus, we can choose $\sigma_{i}^{2}>0$, i=1,…,l large enough such that if $E\left[X_{i}^{2}\right]>\sigma_{i}^{2}$ for some of *i*, then $F_{0}\left(\left(P_{X_{i}}\right)\right)<\sup_{\left(P_{X_{i}}\right)}F_{0}\left(\left(P_{X_{i}}\right)\right)$, irrespective of the choices of $P_{X_{1}},\ldots ,P_{X_{i-1}},P_{X_{i+1}},\ldots ,P_{X_{l}}$. Then, these $\sigma_{1},\ldots ,\sigma_{l}$ are as desired in the proposition. ☐

The non-degenerate assumption ensures that the supremum is achieved:

**Proposition** **2.** *Under Assumption 1,*

*1*. *For any ${\left(P_{X_{i}}\right)}_{i=1}^{l}$, the infimum in* (75) *is attained by some Borel $P_{X}$.*
*2*. *If ${\left(P_{Y_{j}|X^{l}}\right)}_{j=1}^{m}$ are non-degenerate (Definition 3), then the supremum in* (76) *is achieved by some Borel ${\left(P_{X_{i}}\right)}_{i=1}^{l}$.*

The proof of Proposition 2 is given in Appendix E. After taking care of the existence of the extremizers, we get into the tensorization properties, which are the crux of the proof:

**Lemma** **1.** *Fix $\left(P_{X_{i}^{\left(1\right)}}\right)$, $\left(P_{X_{i}^{\left(2\right)}}\right)$, $\left(\mu_{j}\right)$, $\left(T_{j}\right)$, $\left(c_{j}\right)\in {\left[0,\infty \right)}^{m}$, and let $S_{j}$ be induced by coordinate projections. Then:*

(78) $$\begin{array}{c} \underset{P_{X^{\left(1,2\right)}}:S_{i}^{*⊗2}P_{X^{\left(1,2\right)}}=P_{X_{i}^{\left(1\right)}}\times P_{X_{i}^{\left(2\right)}}}{\inf}\sum_{j=1}^{m}c_{j}D\left(P_{Y_{j}^{\left(1,2\right)}}∥\mu_{j}^{⊗2}\right)=\sum_{t=1,2}\sum_{j=1}^{m}c_{j}\underset{P_{X^{\left(t\right)}}:S_{i}^{*}P_{X^{\left(t\right)}}=P_{X_{i}^{\left(t\right)}}}{\inf}D\left(P_{Y_{j}^{\left(t\right)}}∥\mu_{j}\right) \end{array}$$

*where for each j,*

(79) $$\begin{array}{c} P_{Y_{j}^{\left(1,2\right)}}:=T_{j}^{*⊗2}P_{X^{\left(1,2\right)}} \end{array}$$

*on the left side and:*

(80) $$\begin{array}{c} P_{Y_{j}^{\left(t\right)}}:=T_{j}^{*⊗2}P_{X^{\left(t\right)}} \end{array}$$

*on the right side, t=1,2.*

**Proof.**  We only need to prove the nontrivial ≥ part. For any $P_{X^{\left(1,2\right)}}$ on the left side, choose $P_{X^{\left(t\right)}}$ on the right side by marginalization. Then:

(81) $$\begin{array}{cc} D\left(P_{Y_{j}^{\left(1,2\right)}}∥\mu_{j}^{⊗2}\right)-\sum_{t}D\left(P_{Y_{j}^{\left(t\right)}}∥\mu_{j}\right) & =I(Y_{j}^{\left(1\right)};Y_{j}^{\left(2\right)})\\ & \geq 0 \end{array}$$

for each *j*. ☐

We are now ready to show the main result of this section.

**Proof of Theorem 8.** 

1. Assume that $\left(P_{X_{i}^{\left(1\right)}}\right)$ and $\left(P_{X_{i}^{\left(2\right)}}\right)$ are maximizers of $F_{0}$ (possibly equal). Let $P_{X_{i}^{1,2}}:=P_{X_{i}^{\left(1\right)}}\times P_{X_{i}^{\left(2\right)}}$. Define:
  (82) $$\begin{array}{cc} X^{+} & :=\frac{1}{\sqrt{2}}\left(X^{\left(1\right)}+X^{\left(2\right)}\right); \end{array}$$
  (83) $$\begin{array}{cc} X^{-} & :=\frac{1}{\sqrt{2}}\left(X^{\left(1\right)}-X^{\left(2\right)}\right). \end{array}$$
  Define $\left(Y_{j}^{+}\right)$ and $\left(Y_{j}^{-}\right)$ analogously. Then, $Y_{j}^{+}|\left\{X^{+}=x^{+},X^{-}=x^{-}\right\}∼Q_{Y_{j}|X=x^{+}}$ is independent of $x^{-}$, and $Y_{j}^{-}|\left\{X^{+}=x^{+},X^{-}=x^{-}\right\}∼Q_{Y_{j}|X=x^{-}}$ is independent of $x^{+}$.
2. Next, we perform the same algebraic expansion as in the proof of tensorization:
  (84) $$\begin{array}{cc} \sum_{t=1}^{2}F_{0}\left({\left(P_{X_{i}^{\left(t\right)}}\right)}_{i=1}^{l}\right) & =\underset{P_{X^{\left(1,2\right)}}:S_{j}^{*⊗2}P_{X^{\left(1,2\right)}}=P_{X_{j}^{\left(1,2\right)}}}{\inf}\sum_{j}c_{j}D\left(P_{Y_{j}^{\left(1,2\right)}}∥\mu_{j}^{⊗2}\right)-\sum_{i}b_{i}D\left(P_{X_{i}^{\left(1,2\right)}}∥\nu_{i}^{⊗2}\right) \end{array}$$
  (85) $$\begin{array}{c} =\underset{P_{X^{+}X^{-}}:S_{j}^{*⊗2}P_{X^{+}X^{-}}=P_{X_{j}^{+}X_{j}^{-}}}{\inf}\sum_{j}c_{j}D\left(P_{Y_{j}^{+}Y_{j}^{-}}∥\mu_{j}^{⊗2}\right)-\sum_{i}b_{i}D\left(P_{X_{i}^{+}X_{i}^{-}}∥\nu_{i}^{⊗2}\right)\\ \leq \underset{P_{X^{+}X^{-}}:S_{j}^{*⊗2}P_{X^{+}X^{-}}=P_{X_{j}^{+}X_{j}^{-}}}{\inf}\sum_{j}c_{j}\left[D\left(P_{Y_{j}^{+}}∥\mu_{j}\right)+D(P_{Y_{j}^{-}|X^{+}}∥\mu_{j}\left|P_{X^{+}}\right)\right] \end{array}$$
  (86) $$\begin{array}{c} \;-\sum_{i}b_{i}\left[D\left(P_{X_{i}^{+}}∥\nu_{i}\right)+D(P_{X_{i}^{-}|X_{i}^{+}}∥\nu_{i}\left|P_{X_{i}^{+}}\right)\right] \end{array}$$
  (87) $$\begin{array}{c} \leq \sum_{j}c_{j}\left[D\left(P_{Y_{j}^{+}}^{⋆}∥\mu_{j}\right)+D(P_{Y_{j}^{-}|X^{+}}^{⋆}∥\mu_{j}\left|P_{X^{+}}^{⋆}\right)\right]\\ \;-\sum_{i}b_{i}\left[D\left(P_{X_{i}^{+}}^{⋆}∥\nu_{i}\right)+D(P_{X_{i}^{-}|X^{+}}^{⋆}∥\nu_{i}\left|P_{X^{+}}^{⋆}\right)\right] \end{array}$$
  (88) $$\begin{array}{c} =F_{0}\left({\left(P_{X_{i}^{+}}^{⋆}\right)}_{i=1}^{l}\right)+\int F_{0}\left({\left(P_{X_{i}^{-}|X^{+}}^{⋆}\right)}_{i=1}^{l}\right)dP_{X^{+}}^{⋆} \end{array}$$
  (89) $$\begin{array}{c} \leq \sum_{t=1}^{2}F_{0}\left({\left(P_{X_{i}^{\left(t\right)}}\right)}_{i=1}^{l}\right) \end{array}$$
  where:
  - (84) uses Lemma 1.
  - (86) is because of the Markov chain $Y_{j}^{+}-X^{+}-Y_{j}^{-}$ (for any coupling).
  - In (87), we selected a particular instance of coupling $P_{X^{+}X^{-}}$, constructed as follows: first, we select an optimal coupling $P_{X^{+}}$ for given marginals $\left(P_{X_{i}^{+}}\right)$. Then, for any $x^{+}={\left(x_{i}^{+}\right)}_{i=1}^{l}$, let $P_{X^{-}|X^{+}=x^{+}}$ be an optimal coupling of $\left(P_{X_{i}^{-}|X_{i}^{+}=x_{i}^{+}}\right)$ (for a justification that we can select optimal coupling $P_{X^{-}|X^{+}=x^{+}}$ in a way that $P_{X^{-}|X^{+}}$ is indeed a regular conditional probability distribution, see [7]). With this construction, it is apparent that $X_{i}^{+}-X^{+}-X_{i}^{-}$, and hence:
    (90) $$\begin{array}{c} D(P_{X_{i}^{-}|X_{i}^{+}}∥\nu_{i}|P_{X_{i}^{+}})=D(P_{X_{i}^{-}|X^{+}}∥\nu_{i}\left|P_{X^{+}}\right). \end{array}$$
  - (88) is because in the above, we have constructed the coupling optimally.
  - (89) is because $\left(P_{X_{i}}^{\left(t\right)}\right)$ maximizes $F_{0}$, t=1,2.
3. Thus, in the expansions above, equalities are attained throughout. Using the differentiation technique as in the case of forward inequality, for almost all $\left(b_{i}\right)$, $\left(c_{j}\right)$, we have:
  (91) $$\begin{array}{cc} D(P_{X_{i}^{-}|X_{i}^{+}}∥\nu_{i}|P_{X_{i}^{+}}) & =D(P_{X_{i}^{+}}∥\nu_{i}) \end{array}$$
  (92) $$\begin{array}{c} =D(P_{X_{i}^{-}}∥\nu_{i}),\;\forall i \end{array}$$
  where (92) is because by symmetry, we can perform the algebraic expansions in a different way to show that $\left(P_{X_{i}^{-}}\right)$ is also a maximizer of $F_{0}$. Then, $I\left(X_{i}^{+};X_{i}^{-}\right)=D(P_{X_{i}^{-}|X_{i}^{+}}∥\nu_{i}\left|P_{X_{i}^{+}}\right)-D\left(P_{X_{i}^{-}}∥\nu_{i}\right)=0$, which, combined with $I(X_{i}^{\left(1\right)};X_{i}^{\left(2\right)})$, shows that $X_{i}^{\left(1\right)}$ and $X_{i}^{\left(2\right)}$ are Gaussian with the same covariance. Lastly, using Lemma 1 and the doubling trick, one can show that the optimal coupling is also Gaussian.
  ☐

### 4.2. Analysis of Example 1 Using Gaussian Optimality

We note that Example 1 is a rather simple setting, where (17) can be proven by integrating the two sides of (18) and applying the change of variables, noting that the absolute value of the Jacobian equals one. Nevertheless, it is illuminating to give an alternative proof using the Gaussian optimality result, as a proof of concept. In this section, we only give a proof sketch where certain “technicalities” are not justified. Details of the justifications are deferred to Appendix F.

**Proof sketch for the claim in Example 1.**  By duality (Theorem 7), it suffices to prove the corresponding entropic inequality. The Gaussian optimality result in Theorem 8 assumed Gaussian reference measures on the output and non-degenerate forward channels in order to simplify the proof of the existence of minimizers; however, supposing that Gaussian optimality extends beyond those technical conditions, we see that it suffices to prove that for any centered Gaussian $\left(P_{X_{i}}\right)$,

(93) $$\begin{array}{c} \sum_{i=1}^{l}h\left(P_{X_{i}}\right)\leq \underset{P_{X^{l}}}{\sup}\sum_{j=1}^{l}h\left(P_{Y_{j}}\right) \end{array}$$

where the supremum is over Gaussian $P_{X^{l}}$ with the marginals $P_{X_{1}},\ldots ,P_{X_{l}}$ and $Y_{j}:=\sum_{i=1}^{l}m_{ji}X_{i}$. Let $a_{i}:=E\left[X_{i}^{2}\right]$, and choose $P_{X^{l}}=\prod_{i=1}^{l}P_{X_{i}}$; we see that (93) holds if:

(94) $$\begin{array}{c} \sum_{i=1}^{l}\log a_{i}\leq \sum_{j=1}^{l}\log\left(\sum_{i=1}^{l}m_{ji}^{2}a_{i}\right),\;\forall a_{i}>0,\;i=1,\ldots ,l, \end{array}$$

where $\left(a_{i}\right)$ are the eigenvalues and ${\left(\sum_{i=1}^{l}m_{ji}a_{i}\right)}_{i=1}^{l}$ are the diagonal entries of the matrix:

(95) $$\begin{array}{c} M\mathrm{diag}{\left(a_{i}\right)}_{1\leq i\leq l}M^{⊤}. \end{array}$$

Therefore, (94) holds. ☐

A generalization of Example 1 is as follows.

**Proposition** **3.** *For any orthogonal matrix $M:={\left(m_{ji}\right)}_{1\leq j\leq l,1\leq i\leq l}$ with nonzero entries, we claim that there exists a neighborhood U of the uniform probability vector $\left(\frac{1}{l},\ldots ,\frac{1}{l}\right)$, such that for any $\left(b_{1},\ldots ,b_{l}\right)$ and $\left(c_{1},\ldots ,c_{l}\right)$ in U, the best constant D in the FR-BLinequality (16) equals $\exp(H\left(c^{l}\right)-H\left(b^{l}\right))$ where H(·) is the entropy functional.*

The proposition generalizes the claim in Example 1. Indeed, observe that there is no loss of generality in assuming that $\left(b_{1},\ldots ,b_{l}\right)$ and $\left(c_{1},\ldots ,c_{l}\right)$ are probability vectors, since by dimensional analysis, we see that the best constant is infinite unless $\sum_{i=1}^{l}b_{i}=\sum_{j=1}^{l}c_{j}$; and it is also clear that the best constant is invariant when each $b_{i}$ and $c_{j}$ is multiplied by the same positive number. Moreover, any orthogonal matrix can be approximated by a sequence of orthogonal M with nonzero entries, for which the neighborhood U shrinks, but always contains the uniform probability vector $\left(\frac{1}{l},\ldots ,\frac{1}{l}\right)$.

**Proof sketch for Proposition 3.**  Note that along the same lines as (94), the best constant in the FR-BL inequality equals:

(96) $$\begin{array}{c} D=\underset{a^{l}\in \Delta }{\sup}\frac{\prod_{i=1}^{l}a_{i}^{b_{i}}}{\sup_{A⪰0:A_{ii}=a_{i}}\prod_{j=1}^{l}{\left[MAM^{⊤}\right]}_{jj}^{c_{j}}} \end{array}$$

where without loss of generality, we assumed $a^{l}\in \Delta$ is in the probability simplex. We first observe that if the positive semidefinite constraint A⪰0 in (96) were nonexistent, then the sup in the denominator in (96) would equal $\prod_{j=1}^{l}c_{j}^{c_{j}}$, and consequently, (96) would equal $\exp(H\left(c^{l}\right)-H\left(b^{l}\right))$, for any $b^{l},c^{l}\in \Delta$ not necessarily close to the uniform probability vector. Indeed, fixing $A_{ii}=a_{i},i=1,\ldots ,l$, the linear map from the off-diagonal entries to the diagonal entries of ${\mathrm{MAM}}^{⊤}$ is onto the space of *l*-vectors whose entries sum to one; proof of the surjectivity can be reduced to checking the fact that the only diagonal matrix that commutes with M is a multiple of the identity matrix. Then, the sup in the denominator is achieved when ${\left[MAM^{⊤}\right]}_{jj}=c_{j},j=1\ldots l$, which is independent of $a^{l}$.

Next, we argue that the constraint A⪰0 in (96) is not active when $b^{l}$ and $c^{l}$ are close to the uniform vector. Denote by U(t) the set of *l*-vectors whose distance (say in total variation) to the uniform vector $\left(\frac{1}{l},\ldots ,\frac{1}{l}\right)$ is at most *t*. Observe that:

1. There exists t>0 such that for every $a^{l}\in U\left(t\right)$,
  (97) $$\begin{array}{c} \underset{A⪰0:A_{ii}=a_{i}}{\sup}\prod_{j=1}^{l}{\left[MAM^{⊤}\right]}_{jj}=1/l^{l} \end{array}$$
  which follows by continuity and the fact that when $a^{l}$ is uniform, the sup (97) is achieved at the strictly positive definite $A=l^{-1}I$.
2. When $b^{l}=c^{l}=\left(\frac{1}{l},\ldots ,\frac{1}{l}\right)$ is the uniform probability vector, (96) equals one, which is uniquely achieved by $a^{l}=\left(\frac{1}{l},\ldots ,\frac{1}{l}\right)$. To see the uniqueness, take A to be diagonal in the denominator and observe that the denominator is strictly bigger than the numerator when the diagonals of $MAM^{⊤}$ are not a permutation of $a^{l}$. Then, since the extreme value of a continuous functions is achieved on a compact set, we can find ϵ>0 such that:
  (98) $$\begin{array}{c} \frac{\prod_{i=1}^{l}a_{i}^{1/l}}{\sup_{A⪰0:A_{ii}=a_{i}}\prod_{j=1}^{l}{\left[MAM^{⊤}\right]}_{jj}^{1/l}}<1-\epsilon \end{array}$$
  for any $a^{l}\notin U\left(t/2\right)$.
3. Finally, by continuity, we can choose s∈(0,t/2) small enough such that for any $b^{l},c^{l}\in U\left(s\right)$,
  (99) $$\begin{array}{cc} \frac{\prod_{i=1}^{l}a_{i}^{b_{i}}}{\sup_{A⪰0:A_{ii}=a_{i}}\prod_{j=1}^{l}{\left[MAM^{⊤}\right]}_{jj}^{c_{j}}} & <1-\epsilon /2,\;\forall a^{l}\notin U\left(t/2\right); \end{array}$$
  (100) $$\begin{array}{cc} \underset{A⪰0:A_{ii}=a_{i}}{\sup}\prod_{j=1}^{l}{\left[MAM^{⊤}\right]}_{jj}^{c_{j}} & =\underset{A:A_{ii}=a_{i}}{\sup}\prod_{j=1}^{l}{\left[MAM^{⊤}\right]}_{jj}^{c_{j}},\;\forall a^{l}\in U\left(t/2\right); \end{array}$$
  (101) $$\begin{array}{cc} \exp(H\left(c^{l}\right)-H\left(b^{l}\right)) & >1-\epsilon /2. \end{array}$$

Taking the neighborhood U(s) proves the claim. ☐

## 5. Relation to Hypercontractivity and Its Reverses

**Figure 2:**
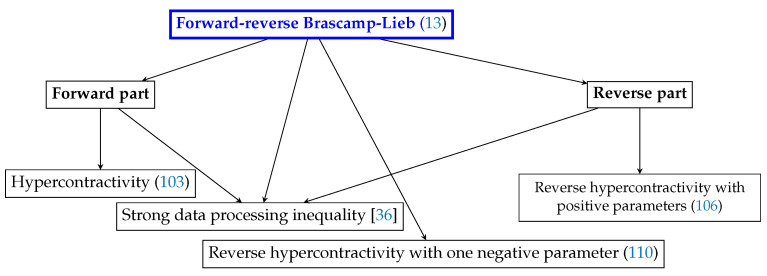
The forward-reverse Brascamp-Lieb inequality generalizes several other functional inequalities/information theoretic inequalities. For more discussions on these relations, see the extended version [7].

As alluded to before and illustrated by Figure 2, the forward-reverse Brascamp-Lieb inequality generalizes several other inequalities from functional analysis and information theory; a more complete discussion on these relationships can be found in [7]. In this section, we focus on hypercontractivity and show how its three cases all follow from Theorem 1. Among these, the case in Section 5.3 can be regarded as an instance of the forward-reverse inequality that cannot be reduced to either the forward or the reverse inequality alone. It is also interesting to note that, from the viewpoint of the forward-reverse Brascamp-Lieb inequality, in each of the three special cases, there ought to be three functions involved in the functional formulation; however, the optimal choice of one function can be computed from the other two. Therefore, the conventional functional formulations of the three cases of hypercontractivity involve only two functions, making it non-obvious to find a unifying inequality.

### 5.1. Hypercontractivity

**Figure 3:**
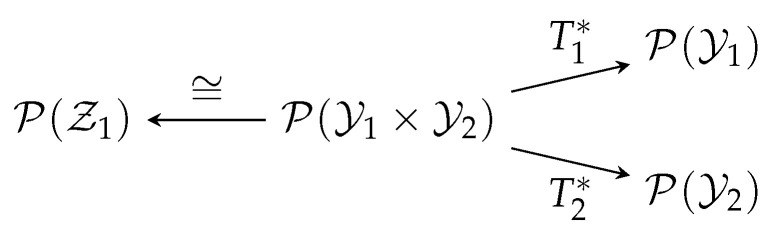
Diagram for hypercontractivity.

Fix a joint probability distribution $Q_{Y_{1}Y_{2}}$ and nonnegative continuous functions $F_{1}$ and $F_{2}$ on $Y_{1}$ and $Y_{2}$, respectively, both bounded away from zero. In Theorem 1, take l←1, m←2, $b_{1}\leftarrow 1$, d←0, $f_{1}\leftarrow F_{1}^{\frac{1}{c_{1}}}$, $f_{2}\leftarrow F_{2}^{\frac{1}{c_{2}}}$, $\nu_{1}\leftarrow Q_{Y_{1}Y_{2}}$, $\mu_{1}\leftarrow Q_{Y_{1}}$, $\mu_{2}\leftarrow Q_{Y_{2}}$. Furthermore, put $Z_{1}=X=\left(Y_{1},Y_{2}\right)$, and let $T_{1}$ and $T_{2}$ be the canonical maps (Definition 2). The measure spaces and the random transformations are as shown in Figure 3.

The constraint (12) translates to:

(102) $$\begin{array}{c} g_{1}\left(y_{1},y_{2}\right)\leq F_{1}\left(y_{1}\right)F_{2}\left(y_{2}\right),\;\forall y_{1},y_{2} \end{array}$$

and the optimal choice of $g_{1}$ is when the equality is achieved. We thus obtain the equivalence between:

(103) $$\begin{array}{c} ∥F_{1}{∥}_{\frac{1}{c_{1}}}{∥F_{2}∥}_{\frac{1}{c_{2}}}\geq E\left[F_{1}\left(Y_{1}\right)F_{2}\left(Y_{2}\right)\right],\;\forall F_{1}\in L^{\frac{1}{c_{1}}}\left(Q_{Y_{1}}\right),\;F_{2}\in L^{\frac{1}{c_{2}}}\left(Q_{Y_{2}}\right) \end{array}$$

and:

(104) $$\begin{array}{c} \forall P_{Y_{1}Y_{2}},\;D\left(P_{Y_{1}Y_{2}}∥Q_{Y_{1}Y_{2}}\right)\geq c_{1}D\left(P_{Y_{1}}∥Q_{Y_{1}}\right)+c_{2}D\left(P_{Y_{2}}∥Q_{Y_{2}}\right). \end{array}$$

By a standard dense-subspace argument, we see that it is inconsequential that $F_{1}$ and $F_{2}$ in (103) are not assumed to be continuous, nor bounded away from zero. It is also easy to see that the nonnegativity of $F_{1}$ and $F_{2}$ is inconsequential for (103).

This equivalence can also be obtained from Theorem 1. By Hölder’s inequality, (103) is equivalent to saying that the norm of the linear operator sending $F_{1}\in L^{\frac{1}{c_{1}}}\left(Q_{Y_{1}}\right)$ to $E\left[F_{1}\left(Y_{1}\right)|Y_{2}=\cdot \right]\in L^{\frac{1}{1-c_{2}}}\left(Q_{Y_{2}}\right)$ does not exceed one. The interesting case is $\frac{1}{1-c_{2}}>\frac{1}{c_{1}}$, hence the name hypercontractivity. The equivalent formulation of hypercontractivity was shown in [44] using a different proof via the method of types/typicality, which requires that $|Y_{1}\left|,\right|Y_{2}|<\infty$. In contrast, the proof based on the nonnegativity of relative entropy removes this constraint, allowing one to prove Nelson’s Gaussian hypercontractivity from the information-theoretic formulation (see [7]).

### 5.2. Reverse Hypercontractivity (Positive Parameters)

By “positive parameters” we mean the $b_{1}$ and $b_{2}$ in (107) are positive.

**Figure 4:**
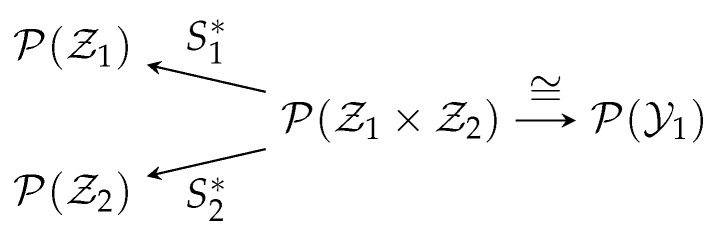
Diagram for reverse hypercontractivity.

Let $Q_{Z_{1}Z_{2}}$ be a given joint probability distribution, and let $G_{1}$ and $G_{2}$ be nonnegative functions on $Z_{1}$ and $Z_{2}$, respectively, both bounded away from zero. In Theorem 1, take l←2, m←1, $c_{1}\leftarrow 1$, d←0, $g_{1}\leftarrow G_{1}^{\frac{1}{b_{1}}}$, $g_{2}\leftarrow G_{2}^{\frac{1}{b_{2}}}$, $\mu_{1}\leftarrow Q_{Z_{1}Z_{2}}$, $\nu_{1}\leftarrow Q_{Z_{1}}$, $\nu_{2}\leftarrow Q_{Z_{2}}$. Furthermore, put $Y_{1}=X=\left(Z_{1},Z_{2}\right)$, and let $S_{1}$ and $S_{2}$ be the canonical maps (Definition 2). The measure spaces and the random transformations are as shown in Figure 4.

Note that the constraint (12) translates to:

(105) $$\begin{array}{c} f_{1}\left(z_{1},z_{2}\right)\geq G_{1}\left(z_{1}\right)G_{2}\left(z_{2}\right),\;\forall z_{1},z_{2}. \end{array}$$

and the equality case yields the optimal choice of $f_{1}$ for (13). By Theorem 1, we thus obtain the equivalence between:

(106) $$\begin{array}{c} ∥G_{1}{∥}_{\frac{1}{b_{1}}}{∥G_{2}∥}_{\frac{1}{b_{2}}}\leq E\left[G_{1}\left(Z_{1}\right)G_{2}\left(Z_{2}\right)\right],\;\forall G_{1},G_{2} \end{array}$$

and:

(107) $$\begin{array}{c} \forall P_{Z_{1}},P_{Z_{2}},\;\exists P_{Z_{1}Z_{2}},\;D\left(P_{Z_{1}Z_{2}}∥Q_{Z_{1}Z_{2}}\right)\leq b_{1}D\left(P_{Z_{1}}∥Q_{Z_{1}}\right)+b_{2}D\left(P_{Z_{2}}∥Q_{Z_{2}}\right). \end{array}$$

Note that in this setup, if $Z_{1}$ and $Z_{2}$ are finite, then Condition (iv) in Theorem 1 is equivalent to $Q_{Z_{1}Z_{2}}\ll Q_{Z_{1}}\times Q_{Z_{2}}$. The equivalent formulations of reverse hypercontractivity were observed in [59], where the proof is based on the method of types.

### 5.3. Reverse Hypercontractivity (One Negative Parameter)

By “one negative parameter” we mean the $b_{1}$ is positive and $-c_{2}$ is negative in (111).

**Figure 5:**
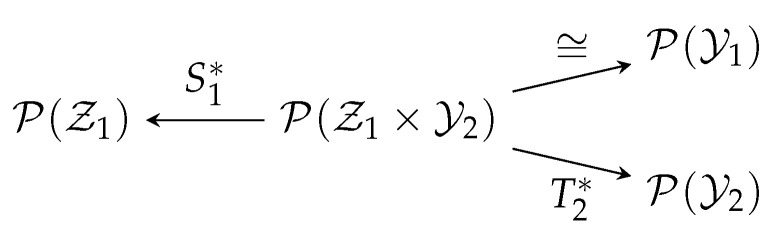
Diagram for reverse hypercontractivity with one negative parameter.

In Theorem 1, take l←1, m←2, $c_{1}\leftarrow 1$, d←0. Let $Y_{1}=X=\left(Z_{1},Y_{2}\right)$, and let $S_{1}$ and $T_{2}$ be the canonical maps (Definition 2). Suppose that $Q_{Z_{1}Y_{2}}$ is a given joint probability distribution, and set $\mu_{1}\leftarrow Q_{Z_{1}Y_{2}}$, $\nu_{1}\leftarrow Q_{Z_{1}}$, $\mu_{2}\leftarrow Q_{Y_{2}}$ in Theorem 1. Suppose that *F* and *G* are arbitrary nonnegative continuous functions on $Y_{2}$ and $Z_{1}$, respectively, which are bounded away from zero. Take $g_{1}\leftarrow G^{\frac{1}{b_{1}}}$, $f_{2}\leftarrow F^{-\frac{1}{c_{2}}}$. in Theorem 1. The measure spaces and the random transformations are as shown in Figure 5.

The constraint (12) translates to:

(108) $$\begin{array}{c} f_{1}\left(z_{1},y_{2}\right)\geq G\left(z_{1}\right)F\left(y_{2}\right),\;\forall z_{1},y_{2}. \end{array}$$

Note that (13) translates to:

(109) $$\begin{array}{c} {∥G∥}_{\frac{1}{b_{1}}}\leq Q_{Y_{2}Z_{1}}\left(f_{1}\right)Q_{Y_{2}}^{c_{2}}\left(F^{-\frac{1}{c_{2}}}\right) \end{array}$$

for all *F*, *G* and $f_{1}$ satisfying (108). It suffices to verify (109) for the optimal choice $f_{1}=GF$, so (109) is reduced to:

(110) $$\begin{array}{c} {∥F∥}_{\frac{1}{-c_{2}}}{∥G∥}_{\frac{1}{b_{1}}}\leq E\left[F\left(Y_{2}\right)G\left(Z_{1}\right)\right],\;\forall F,G. \end{array}$$

By Theorem 1, (110) is equivalent to:

(111) $$\begin{array}{c} \forall P_{Z_{1}},\;\exists P_{Z_{1}Y_{2}},\;D\left(P_{Z_{1}Y_{2}}∥Q_{Z_{1}Y_{2}}\right)\leq b_{1}D\left(P_{Z_{1}}∥Q_{Z_{1}}\right)+\left(-c_{2}\right)D\left(P_{Y_{2}}∥Q_{Y_{2}}\right). \end{array}$$

Inequality (110) is called reverse hypercontractivity with a negative parameter in [45], where the entropic version (111) is established for $|Z_{1}\left|,\right|Y_{2}|<\infty$ using the method of types. Multiterminal extensions of (110) and (111) (called the reverse Brascamp-Lieb type inequality with negative parameters in [45]) can also be recovered from Theorem 1 in the same fashion, i.e., we move all negative parameters to the other side of the inequality so that all parameters become positive.

In summary, from the viewpoint of Theorem 1, the results in Section 5.1, Section 5.2 and Section 5.3 are degenerate special cases, in the sense that in any of the three cases, the optimal choice of one of the functions in (13) can be explicitly expressed in terms of the other functions; hence, this “hidden function” disappears in (103), (106) or (110).

## Appendix A. Recovering Theorem 1 from Theorem 6 as a Special Case

Assume that $P_{X}\to \left(P_{Z_{i}}\right)$ is surjective. Let $1_{Z_{i}}$ denote the constant one function on $Z_{i}$. Define:

(A1) $$\begin{array}{c} C:=\left\{\left(w_{i}\right):w_{i}\in C_{b}\left(Z_{i}\right),\;\sum_{i=1}^{l}\underset{z_{i}}{\inf}w_{i}\left(z_{i}\right)\geq 0\right\}, \end{array}$$

which is a closed convex cone in $C_{b}\left(Z_{1}\right)\times \ldots \times C_{b}\left(Z_{l}\right)$. Given $\left(g_{i}\right)$, we show that $\sum_{i=1}^{l}b_{i}S_{i}\log{\tilde{g}}_{i}\geq \sum_{i=1}^{l}b_{i}S_{i}\log g_{i}$ implies:

(A2) $$\begin{array}{c} {\left(b_{i}\log{\tilde{g}}_{i}-b_{i}\log g_{i}\right)}_{i=1}^{l}\in C. \end{array}$$

Indeed, we can verify that the dual cone:

(A3) $$\begin{array}{cc} C^{*} & :=\left\{\left(\pi_{i}\right):\sum_{i=1}^{l}\pi_{i}\left(w_{i}\right)\geq 0,\;\forall \left(w_{i}\right)\in C\right\}\\ & =\left\{\lambda (P_{Z_{1}},\ldots ,P_{Z_{l}}):\lambda \geq 0\right\}. \end{array}$$

Under the surjectivity assumption, we see:

(A4) $$\begin{array}{c} \sum_{i=1}^{l}\pi_{i}\left(b_{i}\log{\tilde{g}}_{i}-b_{i}\log g_{i}\right)\geq 0,\;\forall \left(\pi_{i}\right)\in C^{*}. \end{array}$$

Now, if (A2) is not true, by the Hahn-Banach theorem (Theorem 5), we find $\pi_{i}\in M\left(Z_{i}\right)$, i=1,…,l such that:

(A5) $$\begin{array}{c} \sum_{i=1}^{l}\pi_{i}\left(b_{i}\log{\tilde{g}}_{i}-b_{i}\log g_{i}\right)<\underset{(w_{i})\in C}{\inf}\sum_{i=1}^{l}\pi_{i}\left(w_{i}\right) \end{array}$$

so the right side of (A5) is not −∞. Since C is a cone containing the origin, the right side of (A5) hence must be nonnegative, and we conclude that $\left(\pi_{i}\right)\in C^{*}$. However, then, (A5) contradicts (A4).

## Appendix B. Existence of Weakly-Convergent Couplings

This section proves an auxiliary result which will be used in Appendix C.

**Lemma** **A1.**
*Suppose that for each i=1,…,l, $P_{X_{i}}$ is a Borel measure on R and $P_{X_{i}}^{\left(n\right)}$ converges weakly to some absolutely continuous (with respect to the Lebesgue measure) $P_{X_{i}}$ as n→∞. If $P_{X}$ is a coupling of ${\left(P_{X_{i}}\right)}_{1\leq i\leq l}$, then, upon extraction of a subsequence, there exist couplings $P_{X}^{\left(n\right)}$ for ${\left(P_{X_{i}}^{\left(n\right)}\right)}_{1\leq i\leq l}$ that converge weakly to $P_{X}$ as n→∞.*

**Proof.** For each integer k≥1, define the random variable $W_{i}^{\left[k\right]}:=\phi_{k}\left(X_{i}\right)$ where $\phi_{k}:R\to R\cup \left\{e\right\}$ is the following “dyadic quantization function”:

(A6) $$\begin{array}{c} \phi_{k}:x\mapsto \left\{\begin{array}{cc} \left\lfloor 2^{k}x\right\rfloor & \left|x\right|\leq k,\;x\notin 2^{-k}Z;\\ e & \mathrm{otherwise}, \end{array}\right. \end{array}$$

and let $W^{\left[k\right]}:={\left(W_{i}^{\left[k\right]}\right)}_{i=1}^{l}$. Denote by $W^{\left[k\right]}:=\left\{-k2^{k},\ldots ,k2^{k}-1,e\right\}$ the set from which $W_{i}^{\left[k\right]}$ takes values. Note that since $P_{X_{i}}$ is assumed to be absolutely continuous, the set of “dyadic points” has measure zero:

(A7) $$\begin{array}{c} P_{X_{i}}\left(\overset{\infty }{\underset{k=1}{⋃}}2^{-k}Z\right)=0,\;i=1,\ldots ,l. \end{array}$$

Since $P_{X_{i}}^{\left(n\right)}\to P_{X_{i}}$ weakly and the assumption in the preceding paragraph precluded any positive mass on the quantization boundaries under $P_{X_{i}}$, for each k≥1, there exists some $n:=n_{k}$ large enough such that:

(A8) $$\begin{array}{cc} P_{W_{i}^{\left[k\right]}}^{\left(n\right)}\left(w\right) & \geq \left(1-\frac{1}{k}\right)P_{W_{i}^{\left[k\right]}}\left(w\right), \end{array}$$

for each *i* and $w\in W^{\left[k\right]}$. Now, define a coupling $P_{W^{\left[k\right]}}^{\left(n\right)}$ compatible with the ${\left(P_{W_{i}^{\left[k\right]}}^{\left(n\right)}\right)}_{i=1}^{l}$ induced by ${\left(P_{X_{i}}^{\left(n\right)}\right)}_{i=1}^{l}$, as follows:

(A9) $$\begin{array}{c} P_{W^{\left[k\right]}}^{\left(n\right)}:=\left(1-\frac{1}{k}\right)P_{W^{\left[k\right]}}+k^{l-1}\prod_{i=1}^{l}\left(P_{W_{i}^{\left[k\right]}}^{\left(n\right)}-\left(1-\frac{1}{k}\right)P_{W_{i}^{\left[k\right]}}\right). \end{array}$$

Observe that (A9) is a well-defined probability measure because of (A8) and indeed has marginals ${\left(P_{W_{i}^{\left[k\right]}}^{\left(n\right)}\right)}_{i=1}^{l}$. Moreover, by the triangle inequality, we have the following bound on the total variation distance:

(A10) $$\begin{array}{c} \left|P_{W^{\left[k\right]}}^{\left(n\right)}-P_{W^{\left[k\right]}}\right|\leq \frac{2}{k}. \end{array}$$

Next, construct $P_{X}^{\left(n\right)}$ (we use ${P|}_{A}$ to denote the restriction of a probability measure *P* on measurable set A, that is ${P|}_{A}\left(B\right):=P\left(A\cap B\right)$ for any measurable B):

(A11) $$\begin{array}{c} P_{X}^{\left(n\right)}:=\sum_{w^{l}\in W^{\left[k\right]}\times \ldots \times W^{\left[k\right]}}\frac{P_{W^{\left[k\right]}}^{\left(n\right)}\left(w^{l}\right)}{\prod_{i=1}^{l}P_{W_{i}^{\left[k\right]}}^{\left(n\right)}\left(w_{i}\right)}\prod_{i=1}^{l}P_{X_{i}}^{\left(n\right)}{|}_{\phi_{k}^{-1}\left(w_{i}\right)}. \end{array}$$

Observe that $P_{X}^{\left(n\right)}$ defined in (A11) is compatible with the $P_{W^{\left[k\right]}}^{\left(n\right)}$ defined in (A9) and indeed has marginals ${\left(P_{X_{i}}^{\left(n\right)}\right)}_{i=1}^{l}$. Since $n:=n_{k}$ can be made increasing in *k*, we have constructed the desired sequence ${\left(P_{X}^{\left(n_{k}\right)}\right)}_{k=1}^{\infty }$ converging weakly to $P_{X}$. Indeed, for any bounded open dyadic cube (that is, a cube whose corners have coordinates being multiples of $2^{-k}$ where *k* is some integer) A, using (A10) and the assumption (A7), we conclude:

(A12) $$\begin{array}{c} \underset{k\to \infty }{lim inf}P_{X}^{\left(n_{k}\right)}\left(A\right)\geq P_{X}\left(A\right). \end{array}$$

Moreover, since bounded open dyadic cubes form a countable basis of the topology in $R^{l}$, we see that (A12) actually holds for any open set A. By writing A as a countable union of dyadic cubes, using the continuity of measure to pass to a finite disjoint union, and then apply (A12), as desired. ☐

## Appendix C. Upper Semicontinuity of the Infimum

Using Lemma A1 in Appendix B, we prove the following result, which will be used in Appendix E.

**Corollary** **A1.**
*Consider non-degenerate $\left(P_{Y_{j}|X}\right)$. For each n≥1, i=1,…,l, $P_{X_{i}}^{\left(n\right)}$ is a Borel measure on R, whose second moment is bounded by $\sigma_{i}^{2}<\infty$. Assume that $P_{X_{i}}^{\left(n\right)}$ converges to some absolutely continuous $P_{X_{i}}^{⋆}$ for each i. Then:*

(A13) $$\begin{array}{c} \underset{n\to \infty }{lim sup}\underset{P_{X}:S_{i}^{*}P_{X}=P_{X_{i}}^{\left(n\right)}}{\inf}\sum_{j=1}^{m}c_{j}D\left(T_{j}^{*}P_{X}∥\mu_{j}\right)\leq \underset{P_{X}:S_{i}^{*}P_{X}=P_{X_{i}}^{⋆}}{\inf}\sum_{j=1}^{m}c_{j}D\left(T_{j}^{*}P_{X}∥\mu_{j}\right). \end{array}$$

**Proof.** By passing to a convergent subsequence, we may assume that the limit on the left side of (A13) exists. For any coupling $P_{X}^{⋆}$ of $\left(P_{X_{i}}^{⋆}\right)$, by invoking Lemma A1 and passing to a subsequence, we find a sequence of couplings $P_{X}^{\left(n\right)}$ of $\left(P_{X_{i}}^{\left(n\right)}\right)$ that converges weakly to $P_{X}^{⋆}$. It is known that under a moment constraint, the differential entropy of the output distribution of a non-degenerate Gaussian channel enjoys weak continuity in the input distribution (see, e.g., [48] Proposition 18, [60] Theorem 7, or [61] Theorem 1 and Theorem 2). Thus:

(A14) $$\begin{array}{c} \lim_{n\to \infty }\sum_{j=1}^{m}c_{j}D\left(T_{j}^{*}P_{X}^{\left(n\right)}∥\mu_{j}\right)=\sum_{j=1}^{m}c_{j}D\left(T_{j}^{*}P_{X}∥\mu_{j}\right) \end{array}$$

and (A13) follows since $P_{X}^{⋆}$ was arbitrarily chosen. ☐

## Appendix D. Weak Semicontinuity of Differential Entropy under a Moment Constraint

This section proves the following result, which will be used in Appendix E.

**Lemma** **A2.**
*Suppose $\left(P_{X_{n}}\right)$ is a sequence of distributions on $R^{d}$ converging weakly to $P_{X^{⋆}}$, and:*

(A15) $$\begin{array}{c} E[X_{n}X_{n}^{⊤}]⪯\Sigma \end{array}$$

*for all n. Then*

(A16) $$\begin{array}{c} \underset{n\to \infty }{lim sup}h\left(X_{n}\right)\leq h\left(X^{⋆}\right). \end{array}$$

**Remark** **A1.**
*The result fails without the condition (A15). Furthermore, related results when the weak convergence is replaced with pointwise convergence of density functions and certain additional constraints were shown in [61] (Theorem 1 and Theorem 2) (see also the proof of [48] (Theorem 5)). Those results are not applicable here since the density functions of $X_{n}$ do not converge pointwise. They are applicable for the problems discussed in [48] because the density functions of the output of the Gaussian random transformation enjoy many nice properties due to the smoothing effect of the “good kernel”.*

**Proof.** It is well known that in metric spaces and for probability measures, the relative entropy is weakly lower semicontinuous (cf. [58]). This fact and a scaling argument immediately show that, for any r>0,

(A17) $$\begin{array}{c} \underset{n\to \infty }{lim sup}h(X_{n}|∥X_{n}∥\leq r)\leq h(X^{⋆}|∥X^{⋆}∥\leq r). \end{array}$$

Let $p_{n}\left(r\right):=P[∥X_{n}∥>r]$, then (A15) implies:

(A18) $$\begin{array}{c} E[XX^{⊤}|∥X_{n}∥>r]\leq \frac{1}{p_{n}\left(r\right)}\Sigma . \end{array}$$

Therefore, since the Gaussian distribution maximizes differential entropy given a second moment upper bound, we have:

(A19) $$\begin{array}{c} h(X_{n}|∥X_{n}∥>r)\leq \frac{1}{2}\log\frac{{\left(2\pi \right)}^{d}e\left|\Sigma \right|}{p_{n}\left(r\right)}. \end{array}$$

Since $\lim_{r\to \infty }\sup_{n}p_{n}\left(r\right)=0$ by (A15) and due to Chebyshev’s inequality, (A19) implies that:

(A20) $$\begin{array}{c} \lim_{r\to \infty }\underset{n}{\sup}p_{n}\left(r\right)h(X_{n}|∥X_{n}∥>r)=0. \end{array}$$

The desired result follows from (A17), (A20) and the fact that:

(A21) $$\begin{array}{c} h\left(X_{n}\right)=p_{n}\left(r\right)h(X_{n}|∥X_{n}∥>r)+\left(1-p_{n}\left(r\right)\right)h(X_{n}|∥X_{n}∥\leq r)+h\left(p_{n}\left(r\right)\right). \end{array}$$

☐

## Appendix E. Proof of Proposition 2

1. For any ϵ>0, by the continuity of measure, there exists K>0 such that:
  (A22) $$\begin{array}{c} P_{X_{i}}\left(\left[-K,K\right]\right)\geq 1-\frac{\epsilon }{l},\;i=1,\ldots ,l. \end{array}$$
  By the union bound,
  (A23) $$\begin{array}{c} P_{X}\left({\left[-K,K\right]}^{l}\right)\geq 1-\epsilon \end{array}$$
  wherever $P_{X}$ is a coupling of $\left(P_{X_{i}}\right)$. Now, let $P_{X}^{\left(n\right)}$, n=1,2,… be such that:
  (A24) $$\begin{array}{c} \lim_{n\to \infty }\sum_{j=1}^{m}c_{j}D\left(P_{Y_{j}}^{\left(n\right)}∥\mu_{j}\right)=\underset{P_{X}}{\inf}\sum_{j=1}^{m}c_{j}D\left(P_{Y_{j}}∥\mu_{j}\right) \end{array}$$
  where $P_{Y_{j}}:=T_{j}^{*}P_{X}$, j=1,…,m. The sequence $\left(P_{X}^{\left(n\right)}\right)$ is tight by (A23). Thus, invoking the Prokhorov theorem and by passing to a subsequence, we may assume that $\left(P_{X}^{\left(n\right)}\right)$ converges weakly to some $P_{X}^{⋆}$. Therefore, $P_{Y_{j}}^{\left(n\right)}$ converges to $P_{Y_{j}}^{⋆}$ weakly, and by the semicontinuity property in Lemma A2, we have:
  (A25) $$\begin{array}{c} \sum_{j=1}^{m}c_{j}D\left(P_{Y_{j}}^{⋆}∥\mu_{j}\right)\leq \lim_{n\to \infty }\sum_{j=1}^{m}c_{j}D\left(P_{Y_{j}}^{\left(n\right)}∥\mu_{j}\right) \end{array}$$
  establishing that $P_{X}^{⋆}$ is an infimizer.
2. Suppose ${\left(P_{X_{i}}^{\left(n\right)}\right)}_{1\leq i\leq l,n\geq 1}$ is such that $E\left[X_{i}^{2}\right]\leq \sigma_{i}^{2}$, $X_{i}∼P_{X_{i}}^{\left(n\right)}$, where $\left(\sigma_{i}\right)$ is as in Proposition 1 and:
  (A26) $$\begin{array}{c} \lim_{n\to \infty }F_{0}\left({\left(P_{X_{i}}^{\left(n\right)}\right)}_{i=1}^{l}\right)=\underset{\left(P_{X_{i}}\right):\Sigma_{X_{i}}⪯\sigma_{i}^{2}}{\sup}F_{0}\left({\left(P_{X_{i}}\right)}_{i=1}^{l}\right). \end{array}$$
  The regularization on the covariance implies that for each *i*, ${\left(P_{X_{i}}^{\left(n\right)}\right)}_{n\geq 1}$ is a tight sequence. Thus, upon the extraction of subsequences, we may assume that for each *i*, ${\left(P_{X_{i}}^{\left(n\right)}\right)}_{n\geq 1}$ converges to some $P_{X_{i}}^{⋆}$. We have the moment bound:
  (A27) $$\begin{array}{cc} E[X_{i}^{2}] & =\lim_{K\to \infty }E\left[\min\left\{X_{i}^{2},K\right\}\right] \end{array}$$
  (A28) $$\begin{array}{c} =\lim_{K\to \infty }E\left[\min\left\{{\left(X_{i}^{\left(n\right)}\right)}^{2},K\right\}\right] \end{array}$$
  (A29) $$\begin{array}{c} \leq \sigma_{i}^{2} \end{array}$$
  where $X_{i}∼P_{X_{i}}^{⋆}$ and $X_{i}^{\left(n\right)}∼P_{X_{i}}^{\left(n\right)}$. Then, by Lemma A2,
  (A30) $$\begin{array}{c} \sum_{i}b_{i}D\left(P_{X_{i}}^{⋆}∥\nu_{i}\right)\leq \lim_{n\to \infty }\sum_{i}b_{i}D\left(P_{X_{i}}^{\left(n\right)}∥\nu_{i}\right) \end{array}$$
  Under the covariance regularization and the nondegenerateness assumption, we showed in Proposition 1 that the value of (76) cannot be +∞ or −∞. This implies that we can assume (by passing to a subsequence) that $P_{X_{i}}^{\left(n\right)}\ll \lambda$, i=1,…,l, since otherwise $F(\left(P_{X_{i}}\right))=-\infty$. Moreover, since ${\left(\sum_{j}c_{j}D\left(P_{Y_{j}}^{\left(n\right)}∥\mu_{j}\right)\right)}_{n\geq 1}$ is bounded above under the nondegenerateness assumption, the sequence ${\left(\sum_{i}b_{i}D\left(P_{X_{i}}^{\left(n\right)}∥\nu_{i}\right)\right)}_{n\geq 1}$ must also be bounded from above, which implies, using (A30), that:
  (A31) $$\begin{array}{c} \sum_{i}b_{i}D\left(P_{X_{i}}^{⋆}∥\nu_{i}\right)<\infty . \end{array}$$
  In particular, we have $P_{X_{i}}^{⋆}\ll \lambda$ for each *i*. Now, Corollary A1 shows that:
  (A32) $$\begin{array}{cc} \underset{P_{X}:S_{i}^{*}P_{X}=P_{X_{i}}^{⋆}}{\inf}\sum_{j}c_{j}D\left(T_{j}^{*}P_{X}∥\mu_{j}\right) & \geq \lim_{n\to \infty }\underset{P_{X}:S_{i}^{*}P_{X}=P_{X_{i}}^{\left(n\right)}}{\inf}\sum_{j}c_{j}D\left(T_{j}^{*}P_{X}∥\mu_{j}\right) \end{array}$$
  Thus, (A30) and (A32) show that $\left(P_{X_{i}}^{⋆}\right)$ is in fact a maximizer.

## Appendix F. Gaussian Optimality in Degenerate Cases: A Limiting Argument

This section proves Theorem 2. We first give a proof for the choice of parameters in Example 1, merely for the sake of notational simplicity, and then discuss how to extend the argument.

### Appendix F.1. Proof of the Claim in Example 1

The proof will be based on Theorem 8, which assumes non-degenerate forward channels and Gaussian measures on the output of the reverse channels. To that end, we will adopt an approximation argument. For each j=1,…,l, define the linear operator $T_{j}^{\epsilon }$ by:

(A33) $$\begin{array}{c} \left(T_{j}^{\epsilon }\phi \right)\left(x_{1},\ldots ,x_{l}\right):=E\left[\phi \left(\sum_{i=1}^{l}m_{ji}x_{i}+N_{\epsilon }\right)\right] \end{array}$$

for any measurable function ϕ on R, where $N_{\epsilon }∼N\left(0,\epsilon \right)$. Let $\gamma_{\frac{1}{\epsilon }}:=N\left(0,\epsilon^{-1}\right)$, and note that the density of $\sqrt{\frac{2\pi }{\epsilon }}\gamma_{\frac{1}{\epsilon }}$ converges pointwise to that of the Lebesgue measure.

**Lemma** **A3.** *For any ϵ>0, let $\left(T_{j}^{\epsilon }\right)$ be defined as in* (A33). *Then, for any Borel $P_{X_{i}}\ll \lambda$, i=1,…,l,*

(A34) $$\begin{array}{c} \sum_{i=1}^{l}D\left(P_{X_{i}}∥\gamma_{\frac{1}{\epsilon }}\right)-\frac{l}{2}\log\frac{2\pi }{\epsilon }\geq \underset{P_{X^{l}}:S_{i}^{*}P_{X^{l}}=P_{X_{i}}}{\inf}\left\{-\sum_{j=1}^{l}h\left(T_{j}^{\epsilon *}P_{X^{l}}\right)\right\}. \end{array}$$

**Proof.** By Theorem 8, it suffices to prove (A34) when $P_{X_{i}}$ is Gaussian, and from (A34), it is easy to see that it suffices to prove the case of the centered Gaussian. Let $P_{X_{i}}=N\left(0,a_{i}\right)$, i=1,…,l. We can upper bound the right side of (A34) by taking $P_{X^{l}}=P_{X_{1}}\times P_{X_{l}}$ instead of the infimum, so it suffices to prove that:

(A35) $$\begin{array}{c} \frac{\epsilon }{2}\sum_{i=1}^{l}a_{i}-\frac{1}{2}\sum_{i=1}^{l}\log a_{i}\geq -\frac{1}{2}\sum_{j=1}^{l}\log\left(\sum_{i=1}^{l}m_{ji}^{2}a_{i}+\epsilon \right) \end{array}$$

for any $\epsilon ,a_{1},\ldots ,a_{l}\in \left(0,\infty \right)$. This is implied by the ϵ=0 case, which we proved in (94). ☐

By the duality of the forward-reverse Brascamp-Lieb inequality (Theorem 7) , we conclude from Lemma A3 that:

**Lemma** **A4.**
*For any ϵ>0 and nonnegative continuous $\left(f_{j}\right)$, $\left(g_{i}\right)$ satisfying:*

(A36) $$\begin{array}{c} \sum_{i=1}^{l}\log g_{i}\left(x_{i}\right)\leq \sum_{j=1}^{l}\left(T_{j}^{\epsilon }\log f_{j}\right)\left(x^{l}\right),\;\forall x^{l}\in R^{l}, \end{array}$$

*we have:*

(A37) $$\begin{array}{c} {\left(\frac{2\pi }{\epsilon }\right)}^{\frac{l}{2}}\prod_{i=1}^{l}\int g_{i}d\gamma_{\frac{1}{\epsilon }}\leq \prod_{i=1}^{l}\int f_{j}\left(x\right)dx. \end{array}$$

Now, suppose that the claim in Example 1 is not true; then there are nonnegative continuous $\left(f_{j}\right)$ and $\left(g_{i}\right)$ satisfying (17) while:

(A38) $$\begin{array}{c} \prod_{i=1}^{l}\int g_{i}\left(x\right)dx>\prod_{i=1}^{l}\int f_{j}\left(x\right)dx, \end{array}$$

By the standard approximation argument, we can assume, without loss of generality, that:

(A39) $$\begin{array}{cc} g_{i}\left(x\right) & =0,\;\forall x:|x|\geq R,1\leq i\leq l; \end{array}$$

(A40) $$\begin{array}{cc} f_{j}\left(x\right) & \geq \delta e^{-x^{2}},\;\forall 1\leq j\leq l, \end{array}$$

for some *R* sufficiently large and δ>0 sufficiently small. Note that for any $x^{l}\in {\left[-R,R\right]}^{l}$,

(A41) $$\begin{array}{c} \sum_{i=1}^{l}m_{ji}x_{i}\in \left[-\sqrt{l}R,\sqrt{l}R\right]. \end{array}$$

Since $\log f_{j}$ is uniformly continuous on $\left[-2\sqrt{l}R,2\sqrt{l}R\right]$ for each *j* and since we assumed (A40), we have:

(A42) $$\begin{array}{c} \lim_{\epsilon \to 0}\underset{x^{l}\in {\left[-R,R\right]}^{l}}{\inf}\left\{\sum_{j=1}^{l}\left(T_{j}^{\epsilon }\log f_{j}\right)\left(x^{l}\right)-\sum_{j=1}^{l}\left(T_{j}^{0}\log f_{j}\right)\left(x^{l}\right)\right\}\geq 0. \end{array}$$

However, since we assumed (17) and (A39), we must also have:

(A43) $$\begin{array}{c} \lim_{\epsilon \to 0}\eta_{\epsilon }\geq 0 \end{array}$$

where:

(A44) $$\begin{array}{c} \eta_{\epsilon }:=\underset{x^{l}\in R^{l}}{\inf}\left\{\sum_{j=1}^{l}\left(T_{j}^{\epsilon }\log f_{j}\right)\left(x^{l}\right)-\sum_{i=1}^{l}\log g_{i}\left(x_{i}\right)\right\}. \end{array}$$

Put:

(A45) $$\begin{array}{cc} {\tilde{g}}_{1}^{\epsilon } & :=\exp\left(\eta_{\epsilon }\right)g_{1}, \end{array}$$

(A46) $$\begin{array}{cc} {\tilde{g}}_{i}^{\epsilon } & :=g_{i},\;i=1,\ldots ,l. \end{array}$$

Then, $\left({\tilde{g}}_{i}^{\epsilon }\right)$ and $\left(f_{j}\right)$ satisfy the constraint (A36) for any ϵ>0. By applying the monotone convergence theorem and then Lemma A4,

(A47) $$\begin{array}{cc} \prod_{i=1}^{l}\int g_{i}\left(x_{i}\right)dx_{i} & \leq \lim_{\epsilon \to 0}{\left(\frac{2\pi }{\epsilon }\right)}^{\frac{l}{2}}\prod_{i=1}^{l}\int {\tilde{g}}_{i}^{\epsilon }d\gamma_{\frac{1}{\epsilon }} \end{array}$$

(A48) $$\begin{array}{c} \leq \prod_{i=1}^{l}\int f_{j}\left(x\right)dx \end{array}$$

which violates the hypothesis (A38), as desired.

### Appendix F.2. Proof of Theorem 2

The limiting argument can be extended to the vector case to prove Theorem 2. Specifically, for each j=1,…,m, define $T_{j}^{\epsilon }$ the same as (A33) except that $N_{\epsilon }∼N\left(0,\epsilon I\right)$, where I is the identity matrix whose dimension is clear from the context (equal to $\dim(E^{j})$ here), and let $P_{Y_{j}|X_{1}\ldots X_{l}}^{\epsilon }$ be the dual operator. For each i=1,…,l, let $\nu_{i}^{\epsilon }:={\left(\frac{2\pi }{\epsilon }\right)}^{\frac{1}{2}\dim\left(E_{i}\right)}\cdot N\left(0,\epsilon^{-1}I\right)$, whose density convergences pointwise to that of $\nu_{i}^{0}$, defined as the Lebesgue measure on $E_{i}$. Define:

(A49) $$\begin{array}{c} d^{\epsilon }:=\sup\left\{\sum_{i=1}^{l}b_{i}\log\nu_{i}^{\epsilon }\left(g_{i}\right)-\sum_{j=1}^{m}c_{j}\log\int f_{j}\right\} \end{array}$$

where the supremum is over nonnegative continuous functions $f_{1},\ldots ,f_{m}$ and $g_{1},\ldots ,g_{l}$ such that the summands in (A49) are finite and:

(A50) $$\begin{array}{c} \sum_{i=1}^{l}b_{i}\log g_{i}\left(x_{i}\right)\leq \sum_{j=1}^{m}c_{j}\left(T_{j}^{\epsilon }\log f_{j}\right)\left(x_{1},\ldots ,x_{l}\right),\;\forall x_{1},\ldots ,x_{l}. \end{array}$$

The same limiting argument (A38)–(A48) extended to the vector case shows that:

(A51) $$\begin{array}{c} d^{0}\leq \lim_{\epsilon ↓0}d^{\epsilon }. \end{array}$$

Next, define $F_{0}^{\epsilon }\left(\cdot \right)$ for $\left(\mu_{j}\right)$, $\left(\nu_{i}^{\epsilon }\right)$ and $P_{Y_{j}|X_{1}\ldots X_{l}}^{\epsilon }$, similarly to (75). The entropic⇒functional argument shows that:

(A52) $$\begin{array}{c} d^{\epsilon }\leq \underset{P_{X_{1}},\ldots ,P_{X_{l}}}{\sup}F_{0}^{\epsilon }\left(P_{X_{1}},\ldots ,P_{X_{l}}\right). \end{array}$$

However, Theorem 8 based on the rotational invariance of the Gaussian measure can be extended to the vector case, so for any ϵ>0,

(A53) $$\begin{array}{c} \underset{P_{X_{1}},\ldots ,P_{X_{l}}}{\sup}F_{0}^{\epsilon }\left(P_{X_{1}},\ldots ,P_{X_{l}}\right)=\underset{P_{X_{1}},\ldots ,P_{X_{l}}c.G.}{\sup}F_{0}^{\epsilon }\left(P_{X_{1}},\ldots ,P_{X_{l}}\right), \end{array}$$

where c.G. means that the supremum on the right side is over centered Gaussian measures. The fact that centered distributions exhaust the supremum follows easily from the definition of $F_{0}$. Moreover, from the definitions, it is easy to see that $F_{0}^{\epsilon }$ is monotonically decreasing in ϵ, and in particular:

(A54) $$\begin{array}{c} \underset{P_{X_{1}},\ldots ,P_{X_{l}}c.G.}{\sup}F_{0}^{\epsilon }\left(P_{X_{1}},\ldots ,P_{X_{l}}\right)\leq \underset{P_{X_{1}},\ldots ,P_{X_{l}}c.G.}{\sup}F_{0}^{0}\left(P_{X_{1}},\ldots ,P_{X_{l}}\right). \end{array}$$

To finish the proof with the above chain of inequalities, it only remains to show that the right side of (A54) equals the supremum in (A49) with $\left(f_{j}\right)$ $\left(g_{j}\right)$ taken over center Gaussian functions. This follows by similar steps as the proof of the functional⇒entropic part of Theorem 1. We briefly mention how the idea works: suppose *A* is the linear space defined as the Cartesian product of R and the set of n×n symmetric matrices. Let Λ(·) be the convex functional on *A* defined by:

(A55) $$\begin{array}{cc} \Lambda (r,M) & :=\ln\int \exp_{e}\left(r+x^{⊤}Mx\right)dx \end{array}$$

(A56) $$\begin{array}{c} =\left\{\begin{array}{cc} r+\frac{n}{2}\ln\pi -\frac{1}{2}\ln\left|-M\right| & M⪯0,\\ +\infty & \mathrm{otherwise}. \end{array}\right. \end{array}$$

The dual space of *A* is itself, and $\Lambda^{*}$ is given by:

(A57) $$\begin{array}{c} \Lambda^{*}\left(s,H\right)=\underset{r,\;M⪰0}{\sup}\left\{sr+\mathrm{Tr}\left(H^{⊤}M\right)-\Lambda \left(r,M\right)\right\}. \end{array}$$

Then, $\Lambda^{*}\left(s,H\right)=+\infty$ if s≠1, and:

(A58) $$\begin{array}{cc} \Lambda^{*}\left(1,H\right) & =\underset{M⪯0}{\sup}\left\{\mathrm{Tr}\left(H^{⊤}M\right)-\frac{n}{2}\ln\pi +\frac{1}{2}\ln\left|-M\right|\right\}. \end{array}$$

The supremum in (A58) equals +∞ if H is not positive-semidefinite. However, if H is positive-semidefinite, the supremum equals $-\frac{1}{2}\ln2\pi e\left|H\right|$, which is equal to the relative entropy between N(0,H) and the Lebesgue measure (supremum achieved when $M=-{\left(2H\right)}^{-1}$). Since the proof of Theorem 1, in essence, only uses the duality between convex functionals, the same algebraic steps therein also establish the desired matrix optimization identity.
